# Effects of Perfluorinated Alkyl Substances (PFAS) on Amphibian Body and Liver Conditions: Is Lipid Metabolism Being Perturbed throughout Metamorphosis?

**DOI:** 10.3390/toxics12100732

**Published:** 2024-10-10

**Authors:** Anna Bushong, Maria Sepúlveda, Meredith Scherer, Abigail C. Valachovic, C. Melman Neill, Sophia Horn, Youn Choi, Linda S. Lee, Priyanka Baloni, Tyler Hoskins

**Affiliations:** 1 Department of Forestry and Natural Resources, Purdue University, West Lafayette, IN 47907, USA; marisep@purdue.edu (M.S.);; 2 Faculty of Life Sciences, Universidad Andres Bello, Santiago 8370146, Chile; 3 Department of Agronomy and Environmental & Ecological Engineering, Interdisciplinary Ecological Sciences and Engineering, Purdue University, West Lafayette, IN 47907, USA; choi226@purdue.edu (Y.C.); lslee@purdue.edu (L.S.L.); 4 College of Health Sciences, Purdue University, West Lafayette, IN 47907, USA; pbaloni@purdue.edu

**Keywords:** perfluorooctanesulfonic acid, perfluorohexanesulfonic acid, perfluorooctanoic acid, perfluorohexanoic acid, *apoa5*, *acox1*

## Abstract

Per- and polyfluoroalkyl substances (PFAS) may interact with peroxisome proliferator activated receptors (PPARs) and alter lipid homeostasis. Using *Xenopus laevis*, we investigated the effect of PFAS on (a) lipid homeostasis and whether this correlated to changes in body and hepatic condition; (b) the expression of hepatic genes regulated by PPAR; and (c) the hepatic lipidome. We chronically exposed tadpoles to 0.5 µg/L of either PFOS, PFHxS, PFOA, PFHxA, a binary mixture of PFOS and PFHxS (0.5 µg/L of each), or a control, from NF stage 52 through metamorphic climax. Growth, development, and survival were not affected, but we detected a sex-specific decrease in body condition at NF 66 (6.8%) and in hepatic condition (16.6%) across metamorphic climax for male tadpoles exposed to PFOS. We observed weak evidence for the transient downregulation of apolipoprotein-V (*apoa5*) at NF 62 in tadpoles exposed to PFHxA. Acyl-CoA oxidase 1 (*acox1*) was downregulated only in males exposed to PFHxS (Ln(Fold Change) = −0.54). We detected PFAS-specific downregulation of structural glycerophospholipids, while semi-quantitative profiling detected the upregulation in numerous glycerophospholipids, sphingomyelins, and diglycerides. Overall, our findings indicate that PFAS can induce sex-specific effects that change across larval development and metamorphosis. We demonstrate that PFAS alter lipid metabolism at environmentally relevant concentrations through divergent mechanisms that may not be related to PPARs, with an absence of effects on body condition, demonstrating the need for more molecular studies to elucidate mechanisms of PFAS-induced lipid dysregulation in amphibians and in other taxa.

## 1. Introduction

Per- and polyfluorinated alkyl substances (PFAS) are ubiquitous environmental pollutants that often bioaccumulate and can elicit toxicity in vertebrate animals via a variety of mechanisms (reviewed by [1,2]). Among these diverse mechanisms, the dysregulation of lipid metabolism is among the most well-supported [2,3]. Along with blood and the kidney, the liver is a primary organ that readily accumulates PFAS [4,5]. Previous studies have indicated that PFAS may perturb lipid homeostasis and that they can alter gene expression in pathways regulating lipid storage, lipid transport, and cholesterol synthesis [6]. Likewise, epidemiological studies of human populations indicate that increased levels of PFAS are associated with a variety of health impacts including altered lipid profiles [3,7], increases in body weight or body condition indices, and increased adiposity [8]; these associations are especially concerning given the strong relationship between adiposity and metabolic disease, including diabetes and cardiovascular disorders [9,10]. Notably, the effects of PFAS across many of these studies show sex as an important mediator of responses. Altogether, evidence suggests that PFAS alter lipid homeostasis in ways that change lipid profiles and body condition.

PFAS are thought to disrupt the ways lipids are stored, transported, transformed, and utilized in cells via multiple mechanisms, but agonism with peroxisome proliferator-activated receptors (PPARs) is among the most well supported [11,12]. Multiple studies across diverse taxa have reasoned that, because many PFAS structurally resemble fatty acids, PFAS might bind PPARs with high affinities (reviewed by [2]), and indeed, PFAS have been shown to bind in vitro with human PPAR subtypes with varying affinities [12,13,14]. Notably, the modulation of lipid homeostasis has been reported as an effect of pharmaceutical PPAR agonists in mice through decreases in fat mass and body weight, leading to steatosis [15,16]. Hepatotoxic damage from PFAS exposure has been connected to PPARα-dependent pathways compared to PPARα-null mice [16,17]. Due to the conservation of PPARs across vertebrates, these findings suggest that PPAR-mediated lipid dysregulation is an important mechanism of PFAS toxicity.

Amphibians are a good system for studying the energetic and ecological consequences of PPAR-mediated effects of PFAS for several reasons. First, from an ecological perspective, lipids are related to amphibian fitness. Lipid stores are often a vital energy source for the successful completion of metamorphosis ([18]; however, see [19]), for survival to reproductive age [20], and for reproduction [21], suggesting that an alteration of larval lipid homeostasis directly influences fitness. Second, available evidence suggests that body condition, measured as the scaled mass index (SMI) [22], is regularly altered following PFAS exposure, often at environmentally relevant concentrations (reviewed by [23]). For example, Hoskins et al. [24] saw reductions in SMI for Northern leopard frogs (*Rana pipiens*) exposed to 0.5 ppb perfluorohexanesulfonic acid (PFHxS) and a 50:50 mixture of perfluorooctanesulfonic acid (PFOS) and PFHxS, summing to 1 ppb. Similarly, Flynn et al. [25] reported reduced SMI in Northern leopard frogs exposed to 10 ppb PFOS, perfluorooctanoic acid (PFOA), PFHxS, and 6:2 fluorotelomer sulfonate (6:2 FTS), while the same exposure regime had no effects on American toads (*Anaxyrus americanus*) and only PFOA, PFHxS, and 6:2 FTS elicited reductions in Eastern tiger salamanders (*Ambystoma tigrium*). Emerging evidence suggests that SMI is positively correlated with energy and lipid reserves in several species of amphibians, including bullfrogs (*Lithobates catesbeianus*) and rough-skinned newts (*Taricha granulosa*)) [26].

There is evidence that PFAS can induce the PPAR-mediated alteration of lipid homeostasis and that this can lead to adverse effects in amphibians. A recent study by Lin et al. [27] observed the dysregulation of lipid metabolism in adult male black-spotted frogs (*Rana nigromaculata*) exposed to PFOS, PFOA, or 6:2 chlorinated polyfluorinated ether (6:2 Cl-PFESA) at 10 ppb for 21 days. They observed a suite of effects across levels of biological organization that were clearly indicative of PPAR-mediated toxicity. Exposure altered metabolism by interacting with PPARs and a variety of other genes and receptors involved in lipid metabolism, but the net effect was an increase in storage lipids (triglycerides and cholesterol) in the blood and liver, and an increased liver mass and hepatosomatic index. The authors were able to link these changes in lipid profiles to hypertrophy and excess fat droplets in liver (i.e., steatosis). In a similar study with the same species, Lin et al. [28] linked altered lipid profiles to other adverse effects, including increased vacuole size and inflammation in hepatocytes and the increased expression of a suite of genes involved in mitigating oxidative stress. Together, these studies highlight that low-dose PFAS exposure can elicit PPAR-mediated toxicity and that these perturbations could be deleterious from an amphibian energetic and fitness standpoint.

Despite important advances in our understanding of how environmentally relevant concentrations of PFAS might influence amphibian fitness through PPAR-mediated lipid dysregulation in adult male black-spotted frogs [27,28,29,30] and adult Chinese toads (*Bufo garagarizans*) [31], how these mechanisms operate in larvae is currently unknown. This is problematic because both the outcomes and the implications of PFAS exposure might be expected to differ across life stages due to differences in lipid regulation and the primary functions of lipids across development. First, primary uptake routes probably differ across these life stages. While the uptake of PFAS in larvae is thought to be driven mostly by gills [32,33], adult aqueous exposures like those conducted by Lin et al. [28] were likely dominated by dermal uptake. Second, the larval period is a unique life-history stage that is a critical period of developmental programming (reviewed by [34]). It is thought that exogenous factors like xenobiotic exposure can alter this programming in irreversible ways that can lead to disease later in life (i.e., the Developmental Origins of Adult Disease Hypothesis [35]). Third, energetic demands vary dramatically as amphibians proceed through development. Embryos rely on maternally derived energy, and many species hatch with remaining yolk, which provides an energy reserve before feeding begins. Conversely, free-swimming larvae feed heavily to fuel growth and development and, if conditions are favorable, to store energy reserves to fuel metamorphosis, a period when tissue reorganization necessitates the cessation of feeding. Metamorphosing amphibians must rely on previously accumulated energy reserves to drive the massive tissue and metabolic reorganization that must occur, which requires a precise orchestration of catabolic and anabolic processes [36]. Perturbations of energetics and associated growth rates during the larval period can affect the energy reserves available for metamorphosis [37] and both size at metamorphosis and lipid levels at metamorphosis are directly linked to post-metamorphic survival and fitness [20]. Energetic demands in the adult stage are thought to differ from both the embryonic and larval stages (reviewed by [38]). These differences in uptake, developmental processes, and physiological states across life stages may explain why Lin et al. [27] found strong evidence for increased lipid accumulation related to PPAR and LXR agonism following PFAS exposure in adult males, while studies of larval amphibians have often documented reduced SMI, possibly driven by reductions in associated lipid content.

In the present study, we hypothesized that PFAS would agonize xPPAR subtypes (α, β, γ), altering the expression of downstream genes directing lipid metabolism and changing the way larval and metamorphosing amphibians store and utilize lipids upon exposure to environmentally relevant concentrations. Specifically, we exposed larval and metamorphic African clawed frogs (*Xenopus laevis*) to two perfluoroalkyl carboxylic acids (PFCA) that differ in chain length (0.5 ppb of PFOA or PFHxA), two perfluoroalkyl sulfonic acids (PFSA) that differ in chain length (0.5 ppb of PFOS or PFHxS), and a binary mixture of PFSA summing to 1 ppb (0.5 ppb of PFOS plus 0.5 ppb PFHxS). By altering lipid metabolism in favor of catabolism, we predicted the following responses to PFAS exposures: lower SMI, altered hepatic condition, the upregulation of associated downstream genes, especially for xPPARα, and xPPARβ, and dysregulation in the profiles of storage lipids (cholesteryl esters (CE), diglycerides (DG), triglycerides (TG)). We also predicted that sex would be an important mediator of the observed effects.

## 2. Materials and Methods

### 2.1. Xenopus laevis Breeding and Husbandry

Larval *X*. *laevis* were bred at the Aquatic Molecular Biology and Analytical Lab of Purdue University (West Lafayette, IN, USA). We injected 750 µL of 500 IU/mL human chorionic gonadotropin (HCG) into the dorsal lymph sac of three females. Egg masses were maintained in culture water (reconstituted reverse osmosis, RO, water to 50 mg/L CaCO_3_ hardness via 9.6 mg/L sodium bicarbonate and 0.132 mL/liter Replenish^®^ using RO water, pH 7–8) with daily partial water changes. All clutches were maintained in 10-gallon aquariums until 4 days post fertilization (dpf) and the highest quality clutch (i.e., largest number of synchronized free-swimming larvae) was selected for further culturing through a common garden period. At 4 dpf, tadpoles were fed Sera Micron^®^ (Sera, Heinsberg, Germany) dissolved in RO water according to feeding protocols (Table S1). The feeding schedule was intended to maintain ad libitum feeding and our target water parameters (dissolved oxygen, ≥3.5 mg/L; pH 7.0–8.5; total ammonia, ≤0.25 mg/L), so, if a scheduled feeding rate increase caused the excess buildup of food from inadequate clearance, feeding levels for all units were returned to the previous bracket until clearance increased. At 5 dpf, larvae were transferred to the Wildlife Ecology Research Facility (WERF, West Lafayette, IN, USA) and acclimated to a 12:12 light cycle and aged well-water (~200 mg/L hardness CaCO_3_, pH 7.5–8.5). Free-swimming larvae were transferred into 15 methanol-rinsed 15 L polypropylene containers in 7.5 L culture water at a density of 40 tadpoles per bin with partial water changes (80%) every 48 h. Tadpoles were developmentally staged using the Nieuwkoop and Faber (NF) staging system [39] and reared in common garden conditions until NF stage 52.

### 2.2. Chemical Selection and Rationale

We selected PFOS, PFHxS, PFOA, and perfluorohexanoic acid (PFHxA) for this study, since they represent the terminal degradation products in the environment for many precursor PFAS and the primary contributors to the sum total PFAS at AFFF-impacted sites [40,41]. The bioactivity of PFAS has been shown to be influenced by two main factors: (a) their carbon chain length and (b) their functional head group [42]. In a recent equilibrium dialysis study, Khazaee et al. [12] identified these four PFAS as having various binding affinities for the human isoforms of PPAR subtypes, further justifying their inclusion to assess this potential mode of toxic action. Therefore, by selecting two long-chain compounds (PFOS, PFOA) and two short-chain compounds (PFHxS, PFHxA) with different functional groups (sulfonic acid vs. carboxylic acid), we were able to assess differences based on these chemical characteristics. For nominal exposure concentrations for single-chemical treatments, we selected 0.5 ppb (μg/L), which represents the upper end of the concentration range for PFAS in surface water near aqueous-fire-fighting foam (AFFF)-impacted field sites [41]. Additionally, we included a binary mixture of PFOS:PFHxS at a total concentration of 1 ppb to represent a common mixture detected at AFFF sites. Notably, the concentration of PFAS in surface waters can vary substantially across the globe and different landscapes, with Podder et al. reporting the median concentration of PFAS in surface water being approximately 135 ng/L [43,44]. Our chosen exposure concentration conceptually represents a possible worse-case scenario for pond-breeding amphibians undergoing their aquatic phase of development in surface water, receiving input from an AFFF or industrially impacted site.

### 2.3. Chemicals, Stock Solution, and Exposure Solution Preparation

PFOS (Lot# BCBR8860V, CAS 1763-23-1), PFHxS (Lot# STBG1147V, CAS 3871-99-6), and PFHxA (Lot# BCCB7797, CAS 307-24-4) were obtained from Sigma Aldrich (St. Louis, MO, USA), while PFOA (Lot# R21120616, CAS 335-67-1) was obtained from Agilent (Santa Clara, CA, USA). We made all the stock solutions using Milli-Q^®^ (Molsheim, France) water in 1 L NalgeneTM (Rochester, NY, USA) high-density polyethylene bottles. The stock solutions for PFOS, PFHxS, and PFOA were prepared by dissolving each compound in 500 mL water to a concentration of 75 parts per million, ppm (i.e., mg/L). Due to PFOA being purchased as free acid, we buffered this stock solution using 1 M NaOH to pH 7.0. Since PFHxA exists in a liquid state at ambient temperature, we incubated 1 mL of PFHxA at 20 °C to use its literature density (1.76 g/mL) to create a 500 mL stock solution at 75 ppm. We dosed 1 L methanol-rinsed deli containers that had 500 mL of aged well-water with their assigned PFAS stock and then added 250 mL of aged well-water for an exposure solution with a final volume of 750 mL at a nominal concentration of 0.5 ppb. A fresh exposure solution was made the morning of each water change and stock solutions were stored at ambient temperature (22 °C ± 1 °C). Stock solutions were sub-aliquoted into 2 mL volumes in methanol-rinsed 5 mL microtubes. Stocks were stirred prior to aliquoting and stocks re-aliquoted from the primary stock periodically throughout the study.

### 2.4. Experiment Initiation and Maintenance

We conducted this experiment at WERF. This study included six experimental treatments: control, PFOS, PFHxS, PFOA, PFHxA, and a binary mixture of PFOS:PFHxS. Animals were lethally sampled during metamorphic climax. Experimental replicates consisted of 1 L methanol-rinsed deli containers holding 750 mL of exposure solution and a single *X*. *laevis* larvae. There were 36 replicates per PFAS treatment, with 12 replicates designed for lethal sampling at each selected developmental stage across three spatial blocks split between two shelf-racks, following a randomized block design. We reared tadpoles under common garden conditions until NF 52, anesthetized using 140 mg/L buffered MS-222, and sorted into 15 L polypropylene containers by stage. Tadpoles were arbitrarily added to deli containers with 250 mL of aged well-water. We initiated the experiment by spatial block, and pre-dosed 500 mL of aged well-water and then poured it in a container with 250 mL of water and a single tadpole. One dummy unit (without a tadpole) with a HOBO (Cape Cod, MA, USA) logger was added per spatial block for continuous temperature monitoring. Pooled water samples (*n* = 3) representing 3 experimental replicates were collected for all treatments at initiation, and again when sampling began for each developmental stage (NF 58, 62, 66). Controls (aged well-water with a tadpole) from each spatial block were monitored to track water quality prior to each water change to ensure target criteria were maintained, including dissolved oxygen (DO) ≥3.5 mg/L, pH 7.0–8.5 and total ammonia ≤0.25 mg/L. Study units were kept at ambient water temperature (21 °C ± 1 °C) with a 12:12 photoperiod. Due to the total number of experimental units (*n* = 216), we performed water changes in a staggered manner, performing 100% water changes every 48 h for each shelf rack by gently transferring tadpoles with an aquarium net. The first water change was performed 24 h after experimental initiation and the shelf-rack was randomly selected. Tadpoles were fed twice per day (~9:00 and ~15:00 EST) (Table S1). At NF 62, *X*. *laevis* tadpoles reach a non-feeding stage and feeding ceased until after the next water change, when larvae were fed once more to confirm non-feeding status and ensure the ad libitum feeding was maintained.

### 2.5. Apical Data and Tissue Collection

After reaching their pre-designated sampling stage, animals were sampled in the order of the randomized block design and euthanized using 3 g/L buffered MS-222. NF stage, date, time, snout–vent length (SVL), and wet body mass were recorded for each tadpole. Digital calipers were used to measure SVL with the tadpole position on its right side (head oriented left). Upon dissection, whole liver mass was also recorded. Tissues were collected referencing the Xenopus Tissue Harvesting protocol [45] collecting the following: liver, which was bisected to preserve one portion in RNAlater^®^ (ThermoFisher Scientific, Waltham, MA, USA) for RT-qPCR and the other flash-frozen for multiple reaction monitoring (MRM) lipidomic profiling, and either tail clips (NF 56–62) or two-outer digits of the right hindfoot (NF 66), flash-frozen for genetic sexing.

### 2.6. PFAS Water Analyses

One water sample representing a pooling of 3 experimental units per treatment was analyzed for PFAS (Table 1). Water samples were transferred to a 1.5 mL glass injection vial along with 230 μL of methanol and 20 µL of 250 ng/mL of mass-labeled PFAS in methanol as an internal standard solution. The mixture was vortexed (1500 rpm for 10 min) and stored at 4 °C until analysis. Laboratory blanks and spiked control samples were prepared concurrently with water and tissue sample batches.

For PFAS quantification in the water samples, a Shimadzu ultraperformance liquid chromatography system with a Triple Quadrupole Liquid Chromatograph Mass Spectrometer (LC/MS/MS) system (Shimadzu LCMS-8040, Columbia, MD, USA) operated in negative—with an electrospray ionization source mode (LC-ESI-MS/MS)—was used in negative ion mode. To prevent potential contamination, a Restek (Bellefont, PA, USA) PFAS Delay Column (5 um, 50 × 2.1 mm) was installed between the autosampler and pump compartments. The analytical column utilized was a Kinetex^®^ 5 μm EVO C18 (Phenomenex, Torrance, CA, USA, 100 Å, LC Column 100 × 2.1 mm) with a guard filter (Phenomenex, KrudKatcher ULTRA HPLC In-Line Filter, 2.0 μm Depth Filter ×0.004 in ID) at a maintained temperature of 40 °C. The injection volume was 10 μL, and a combined flow rate of 0.4 mL/min was used for mobile phase A (20 mM ammonium acetate in water) and B (methanol). The gradient followed a specific pattern, starting at 10% B and increasing to 50% within the first 0.5 min, reaching 99% at 8 min and remaining constant for 5 min, before decreasing back to 10% within 0.5 min, then staying constant for 20 min. The acquired data on scheduled multiple reaction monitoring (MRM) mode was processed using LabSolutions software (ver. 6.115).

### 2.7. RT-qPCR Analysis

RNA extraction: A bisected portion of liver was stored in RNAlater^®^, left at ambient temperature for 48 h, then stored at −20 °C until processing. Total RNA was extracted using Qiagen RNeasy (Germantown, MD, USA) mini-kits according to manufacturer protocols, with modifications recommended for liver tissue. Individual samples provided adequate RNA yield and purity, which were measured using a Nanodrop 8000 spectrophotometer (260/280 = 2.0 ± 0.2) (ThermoFisher, Waltham, MA, USA) prior to storage at −80 °C. After thawing, RNA was treated for DNA contamination with DNase 1 (ThermoFisher, Waltham, MA, USA) immediately prior to cDNA synthesis.

cDNA synthesis: Our biological replicates constituted a bisected portion of a single tadpole liver, amounting to *n* = 9 per PFAS treatment. cDNA synthesis was performed with 200 ng total DNase-treated RNA starting material and SuperScript III reverse transcriptase (Invitrogen, Carlsbad, CA, USA) per manufacturer instructions. Synthesized cDNA was diluted to a working concentration of 2 ng/μL and stored at −20 °C until qPCR analysis.

RT-qPCR assays, primer design and validation: RT-qPCR assays were performed in technical duplicate on a QuantStudio 3 Real-Time qPCR system (ThermoFisher) with iQ SYBR Green SuperMix (Bio-Rad, Hercules, CA, USA) following thermal protocols provided by the manufacturer. RT-qPCR primers for *Xenopus laevis* xPPAR*α*/*β*/*γ*, target genes of interest (*apoa5*, *fabp1*, *acox1*, *pck1*) and reference gene (*sub1*) were sourced from the literature or developed using published NCBI nucleotide data, Primer3Plus software (v3.3.0), and the UCSC genome browser. Primer pair reaction efficiency was determined using serially diluted standard curves, with cDNA derived from NF 48 whole tadpoles (Table S3). Additional information on primer parameters and validation process can be found in the Supplementary Files.

Polymerase Chain Reaction (PCR) for genetic sex determination: Tissues collected for genetic sex were held at −80 °C until analyzed. DNA was extracted using the Qiagen DNeasy^®^ blood and tissue kit (Hilden, Germany) according to manufacturer instructions. Genetic sex was determined using PCR and females identified through the presence of the DM-W gene, which is a W-linked gene necessary for ovary formation in *X. laevis* ZW individuals [46].

### 2.8. Multiple Reaction Monitoring (MRM) Profiling

Lipid extraction and sample preparation for mass spectroscopy: We performed lipid extractions of flash-frozen livers using the Bligh and Dyer protocol [47,48]. Samples were homogenized in double-deionized water (ddH_2_O) in a volume of water that yielded a homogenate concentration equivalent to 1 mg/200 µL. The volume of 200 µL of liver homogenate was transferred to a microtube, and methanol (550 µL) and chloroform with 0.1% butylated hydroxytoluene (250 µL) were added. Samples were incubated at 4 °C for 15 min, followed by an addition of ddH_2_O (250 µL) and more chloroform (250 µL). Samples were centrifuged at 10,000× *g* for 10 min, leading to phase separation with a polar upper phase and a non-polar bottom phase. The bottom layer was retrieved using a micropipette, placed in a new microtube, and the solvent evaporated using a Savant^TM^ SpeedVac^TM^ Vacuum Concentrator (ThermoFisher) at ambient temperature for 1.5 h. Following evaporation, lipid residues were stored at −80 °C until MS analysis. For injection, dried lipid residues were reconstituted with acetonitrile, methanol, and ammonium acetate at 300 mM (6.65:3.00:0.35). For each sample injection, 8 µL of solution, with an internal standard equivalent to 0.8 ng, was injected at a rate of 10 µL/min. The data were acquired on a triple quadrupole mass spectrometer (Agilent QQQ 6410, Santa Clara, CA, USA) utilizing an autosampler (Agilent G1367A 1100 series).

Unlike other lipidomic techniques that rely on liquid chromatography, MRM profiling characterizes the lipidome of a sample as a set of functional groups instead of as individual molecules, by using precursor and neutral loss MS/MS ion scans [49]. MRM profiling can be broken down into three distinct phases: (1) discovery, (2) screening, and (3) identification. The first two phases were utilized for this study. The discovery phase describes the process of selection of MRM transitions (i.e., the precursor and neutral loss MS/MS ion scans), which were based on the database LIPID MAPS^®^, via data collection on MRM transitions on a few representative samples. The lipid and metabolite classes considered included glycerophospholipids (PA, PC, LPC, PE, LPE, PG, PI, PS), acyl-carnitines (CARs), ceramides (CERs), sphingomyelins (SMs), cholesteryl esters (CEs), diglycerides (DGs), and triglycerides (TGs). Across these classes, a total of 3259 MRM transitions were evaluated. Transitions were retained for the screening phase if they had 30% or higher ion counts relative to blank samples, which amounted to 497 MRM transitions. Selected transitions were sorted into four MRM methods (three in positive ion mode, one in negative ion mode) and broadly grouped into structural lipids (PC, LPC, PE, LPE: 203 MRM transitions; Method 1), structural lipids and signaling molecules (CAR, CER, SM, PS, PG, PI: 162 MRM transitions; Method 2), storage lipids (DG, TG, CE: 85 MRM transitions; Method 3), and fatty acids (FFA: 47 MRM transitions; Method 4). To profile each lipid class, one sample injection was performed per method with *n* = 24 per PFAS treatment (*n* = 8 per NF stage/PFAS treatment) (a total of 576 sample injections).

### 2.9. Data Analysis and Statistics

All data analysis and statistics were carried out in R or MetaboAnalyst 5.0 and 6.0 (R version 4.3.1) [50,51].

Morphometric data and time-to-stage analysis: The morphological and developmental endpoints were analyzed using factorial independent analyses of variance (ANOVA). Assumptions for normality and homogeneity of variance were visually assessed utilizing plots of residuals and, for the latter, also through a Levene’s test. After grouping by treatment, developmental stage and genetic sex, morphometric data were checked for extreme outliers, as defined by a 3× interquartile range (IQR) criterion, excluding these experimental units prior to rechecking assumptions using residual plots and proceeding with statistical analysis. For a given endpoint, we defined statistical significance for a developmental group or experimental treatment as a difference of *p* ≤ 0.05 compared to the control group. The time-to-event analysis was conducted using a multivariate Cox proportional hazard model from the “survival” package in R. Each NF stage was investigated separately to determine the association between the developmental time and PFAS treatment while including genetic sex as a covariate.

Scaled Mass Index (SMI): We calculated the scaled mass index (SMI) in accordance with the approach reported by Peig and Green [22] and Flynn et al. [25] (Equation (1)). For this study, the reference animals were from the control treatment by NF stage to ensure neither developmental stage nor treatment effects skewed the average length measurement used to calculate the bSMA scaling coefficient. We also used this approach to calculate scaled hepatic index (SHI) to report alongside the commonly used hepatic somatic index (HSI).

(1) $${\hat{M}}_{i}=M{\left[\frac{L_{0}}{L_{i}}\right]}^{b_{\mathrm{SMA}}}$$

Equation (1): The formula for calculating SMI, where M_i_ and L_i_ represent mass and length for the same animal, respectively; L_0_ represents the average length of the reference group of animals; the b_SMA_ exponent scales the relationship to the reference group; and ${\hat{M}}_{i}$ denotes the SMI for a given animal (i.e., predicted body mass at the average length of the reference group).

Scaled Hepatic Index (SHI) and Liver Somatic Index (HSI): We calculated two hepatic indices (SHI and HSI), to evaluate the effect of PFAS exposure on hepatic condition. HSI (also referred to as liver somatic index, LSI) has been utilized as a bioindicator in aquatic toxicology for hepatomegaly induced by suspected hepatotoxic xenobiotics, particularly in fish. For anurans, the EPA Larval Amphibian Growth and Development Assay (LAGDA) utilizes HSI as an indicator of hormonal activity when evaluating the effects of endocrine-disrupting chemicals, as these compounds may alter liver mass through metabolic disturbance causing changes in lipid content or inflammation [52,53] (Equation (2)). However, HSI does not substantially account for structural reorganization tadpoles undergo during metamorphosis, so the simultaneous evaluation of a hepatic index that has been standardized for structural size offers the opportunity to compare indices. Peig and Green [22] note that their approach for calculating SMI can be applied to standardize the mass of any compartment. Therefore, SHI was calculated in the same manner as SMI (Equation (1)), but whole liver mass was used instead of individual body mass. Both HSI and SHI were analyzed using factorial independent ANOVA after assessment of normality and homogeneity of variance. After grouping by treatment, developmental stage, and sex, these indices were evaluated using a stricter 2× IQR criterion to ensure unit exclusion overtly skewing the treatment mean, excluding these experimental units prior to rechecking assumptions using residual plots and proceeding with statistical analysis.

(2) $$\mathrm{Hepatic}\;\mathrm{Somatic}\;\mathrm{Index}\;\left(\mathrm{HSI}\right)=\frac{\mathrm{Whole}\;\mathrm{Liver}\;\mathrm{Mass}\;\left(g\right)}{\mathrm{Whole}\;\mathrm{Body}\;\mathrm{Mass}\;\left(g\right)}*100\%$$

Equation (2): The formula for calculating HSI, expressing the proportion of liver mass to body mass as a percentage.

qPCR analysis of gene expression: An analysis of gene expression was performed according to the Pfaffl method using calculated relative mRNA for all samples [54] (Equation (3)). Raw Ct data underwent review to exclude unreliable values (Ct > 35). For this study, the calculation of relative mRNA for target genes was carried out by developmental stage relative to the housekeeping gene *sub1*. Prior to the statistical analysis of gene expression by developmental stage, relative mRNA was natural log transformed before using factorial independent ANOVAs to evaluate the effect of PFAS treatment, followed by appropriate post hoc comparisons at a fixed threshold of *p* ≤ 0.05 for statistical significance.

(3) $$\mathrm{Relative}\;\mathrm{mRNA}=\frac{E_{\mathrm{target}}^{\left({\bar{\mathrm{Ct}}}_{\mathrm{RefG}}-{\bar{\mathrm{Ct}}}_{\mathrm{ExpS}}\right)}}{E_{\mathrm{housekeeping}}^{\left({\bar{\mathrm{Ct}}}_{\mathrm{RefG}}-{\bar{\mathrm{Ct}}}_{\mathrm{ExpS}}\right)}}$$

Equation (3): The formula for the calculation of relative mRNA (i.e., Relative Fold Change). E represents the converted primer efficiency of either the target gene or housekeeping gene used for normalization. E is exponentiated to ΔCt, which is computed as the average Ct of the gene in the reference group minus the average Ct of the gene between the technical duplicates of the experimental sample.

MRM profiling analysis: We processed the MRM mass spectrometry data using Microsoft Excel to calculate the relative ion intensity of each lipid class by normalizing the absolute ion intensity of lipids and metabolites to the total ion current of the MRM method to produce a sample’s lipid profile. Prior to the calculation of relative ion intensity, samples were spot-checked across various MRM transitions to identify faulty samples by visualizing ion intensities with bar charts, taking note of samples with ion intensities that dramatically differ (e.g., the sample had an ion intensity signal consistently half of the average of other samples across multiple MRM transitions). Additionally, except for CAR and FFA and due to a lack of internal standards available, we calculated semi-quantitative ion intensity by lipid class normalized to the ion intensity of an internal standard, to investigate whether absolute amounts of lipids changed within a class, apart from the overall lipid composition.

Relative quantities were organized into tables where columns were samples and rows representing MRM transitions for analysis using MetaboAnalyst 5.0 and 6.0 [51]. To investigate differences by PFAS treatment at each NF stage, we used MetaboAnalyst to perform linear models with covariate correction based on the LIMMA package and visualized data with heatmaps, which were also used to check for potential outliers not identified through spot-checking internal standards. Linear models with covariate correction were performed in place of factorial ANOVAs in MetaboAnalyst due to the program directing its utilization for unbalanced groups. For covariates, we utilized spatial blocks, NF stage, and genetic sex. Due to the structure of the interface, the linear model tool returns the adjusted *p*-value for the main effect of treatment, and upon detecting significant treatment effects, the user must specify individual treatment contrasts to obtain an adjusted *p*-value for the contrast. The fixed threshold for statistical significance was a false discovery rate (FDR)-adjusted *p* ≤ 0.05. Due to the exploratory nature of this method, we also report lipid species for a class with weaker evidence (*p* ≤ 0.1) of change based on the FDR.

## 3. Results

### 3.1. Effects of Sub-Chronic PFAS Exposure on Morphometric Endpoints and Time-to-Stage

When considering all stages, we did not observe the main effect of PFAS exposure on mass, SVL, or SMI (Figure S1; Table S4). However, the effect of stage was significant for all three endpoints and proceeded with stage specific analyses.

For NF 58, there was no effect of PFAS exposure on mass or SMI, but weak evidence for an effect on SVL is observed (Table S5). To further assess this weak effect, we performed a Dunnett’s test, which indicated weak evidence for an increase in average SVL (4.85%) for animals exposed to PFOS (Table S6). There was also a small, main effect of genetic sex on SVL, with males being 1.85% longer than females.

For NF 62, we did not observe a main effect of PFAS on mass or SVL. However, we did observe a main effect of PFAS on SMI with weak evidence of an interaction with genetic sex, but a post hoc Dunnett test indicated PFAS did not significantly affect SMI relative to controls (Tables S7 and S8). Given the potential interaction, we also analyzed SMI by genetic sex and found no effect of PFAS on SMI at this stage.

For NF 66, we did not detect a main effect of PFAS on mass, SVL, or SMI (Table S9). We did detect an effect of sex on SVL and SMI, but these changes were small in magnitude: females had a slightly higher average SVL (1.05%) and males had a slightly higher average SMI (1.32%). However, when analyzing sex separately and adjusting for spatial block, there was evidence for an effect of PFAS on the SMI of males with a Dunnett test indicating a 6.79% decrease in SMI following exposure to PFOS (Tables S9 and S10).

For time-to-stage, our Cox proportional hazard analysis indicated that there was no impact of PFAS treatment on the number of days to reach NF 58 (Chi-square = 6.441 ; df = 5; *p* = 0.27), NF 62 (Chi-square = 4.765 ; df = 5; *p* = 0.45), or NF 66 (Chi-square = 2.902 ; df = 5; *p* = 0.72) when accounting for genetic sex (Figure S2).

### 3.2. Effects of Sub-Chronic PFAS Exposure on Hepatic Condition

**Figure 1:**
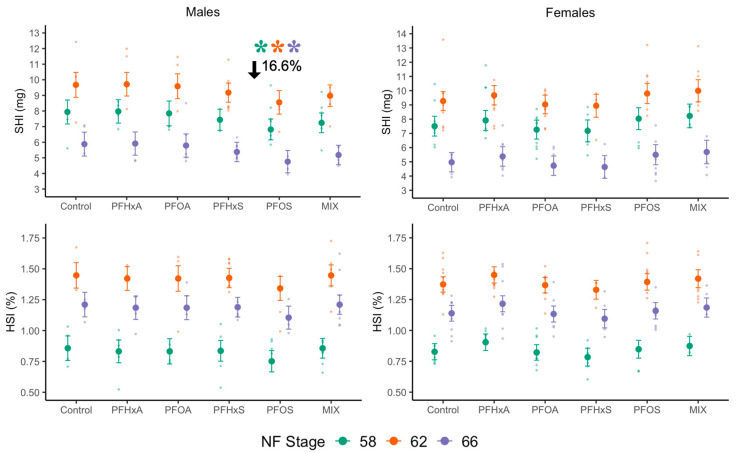
Marginal mean ± 95% C.I. SHI and HSI for *X. laevis* exposed to different PFAS (control, PFHxA, PFHxS, PFOA, PFOS, Mix (PFHxS:PFOS)) across NF stages with raw data displayed in the background. Colors indicate NF stage and asterisks (*) indicates a significant difference between the mean of the pooled groups and the control. Moderate and extreme outliers (2 × IQR criterion) excluded. *n* = 33–36 per PFAS treatment (*n* = 3–9 per sex/NF stage/PFAS treatment).

We did not observe effects of PFAS exposure on HSI when considering genetic sex, but detected a significant interaction between genetic sex and PFAS exposure on SHI (Figure 1; Table S11). There was no significant main effect of PFAS exposure on SHI in females and weak evidence (*p* < 0.1) for a transitory increase in SHI for females at NF 62 (Table S12). For males, there was a significant main effect of PFAS exposure on SHI that was consistent across developmental stages. A Dunnett’s test indicated that male *X. laevis* exposed to PFOS had average SHI 16.6% lower than controls (Figure 1; Tables S11 and S13). In contrast, exposure to the shorter chain PFHxS, PFAS with a carboxylate functional group (PFHxA, PFOA), or a binary mixture of PFAS with sulfonate functional groups (PFHxS:PFOS) had no impact on male *X. laevis* SHI.

### 3.3. Effects of Sub-Chronic PFAS Exposure on Gene Expression

In NF 58 larvae, the expression of acox1, apoa5, fabp1, and pck1 did not significantly change relative to controls across PFAS treatments (Table S14). For NF 62, the expression of acox1 and pck1 did not change relative to controls across PFAS treatments, with variable evidence for the treatment-specific downregulation for apoa5 and fabp1 (Figure S3; Tables S15–S19). There was no significant main effect of PFAS on acox1 or pck1 expression. For apoa5 and fabp1, there was a significant main effect of PFAS treatment (Table S16). A post hoc Dunnett test indicated weak evidence that PFHxA and PFOA downregulated apoa5 gene expression (Table S17). For fabp1, a post hoc Dunnett test also indicated significant downregulation for animals exposed to PFOA. However, the lack of genetic males analyzed for gene expression in the PFOA treatment at NF 62 (*n* = 1) makes those treatment signals less reliable, as only weak evidence was detected when sex is excluded as a covariate (Tables S18 and S19).

In NF 66, the expression of apoa5, fabp1, and pck1 did not significantly change relative to controls across PFAS treatments, but acox1 expression changed in males (Figure S4; Tables S20–S22). There was no significant main effect of PFAS treatment on apoa5, fabp1, or pck1 expression. However, there was weak evidence for an interaction between genetic sex and PFAS treatment for acox1, so we chose to conduct a subsequent analysis by sex and detected downregulation only in males (Table S20). Downregulation was induced focally by PFHxS (Ln(Fold Change) = −0.54, t = −3.9, *p* = 0.005), with weak evidence of downregulation by PFOA (Table S21). These signals were lost without the inclusion of genetic sex (Table S22).

### 3.4. Effects of Sub-Chronic PFAS Exposure on Relative Lipidomic Signatures

**Figure 2:**
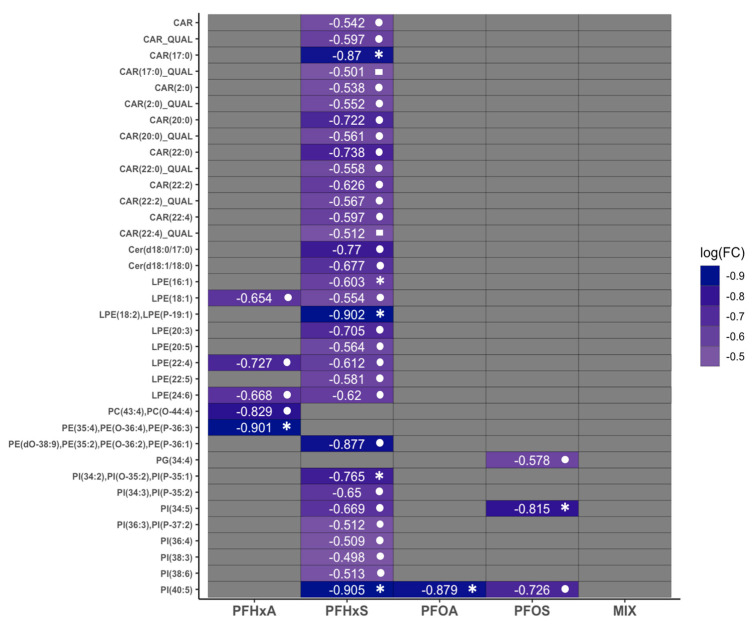
Heat map displaying log2 fold change (logFC) for relative ion intensity of hepatic lipid species and signaling molecules by PFAS treatment, using LIMMA with covariate adjustment (spatial block, developmental stage, genetic sex). Shapes represent FDR-adjusted *p*-values: asterisks, *p* ≤ 0.05; circle, *p* ≤ 0.1; square, *p* ≤ 0.15). Faulty samples identified based on ionization of internal standards and visualization using heatmaps were excluded by lipid class for analysis. *n* = 22–24 per PFAS treatment (*n* = 7–8 per NF stage/PFAS treatment).

We observed changes in the relative abundance of select acyl-carnitines and glycerophospholipids following PFAS exposure, with weak evidence for changes in ceramides (Figure 2; Table S23). We proceeded with an analysis of lipid classes using linear models with covariate adjustment for spatial blocks, NF stage, and genetic sex. For structural glycerophospholipids, we detected the significant downregulation of phosphatidylethanolines (PEs), lysophosphatidylethanolines (LPEs), and phosphatidylinositols. Phosphatidylglycerols (PGs) and phosphatidylserines (PSs) did not change with exposure to PFAS. PEs had one significant MRM transition representative of multiple lipids that was downregulated by PFHxA. An MRM transition representative of multiple PEs was downregulated by PFHxS, but evidence for this contrast was weak upon FDR adjustment (*p* ≤ 0.1). LPE lipids were lower upon exposure to PFHxS. Phosphotidylinositols (PIs) were the only glycerophospholipid that had different lipid species significantly decrease across all four singular-PFAS exposures.

For signaling lipids and metabolites, there was a significant differential lipid detected for acyl-carnitines (CARs) and weak evidence of broader downregulation for PFHxS only. Additionally, there was weak evidence for ceramide (CER) downregulation. There were no changes for storage lipids, including cholesteryl esters (CEs), diglycerides (DGs), and triglycerides (TGs), or free-fatty acids (FFAs).

### 3.5. Effects of Sub-Chronic PFAS Exposure on Semi-Quantitative Lipidomic Signatures

**Figure 3:**
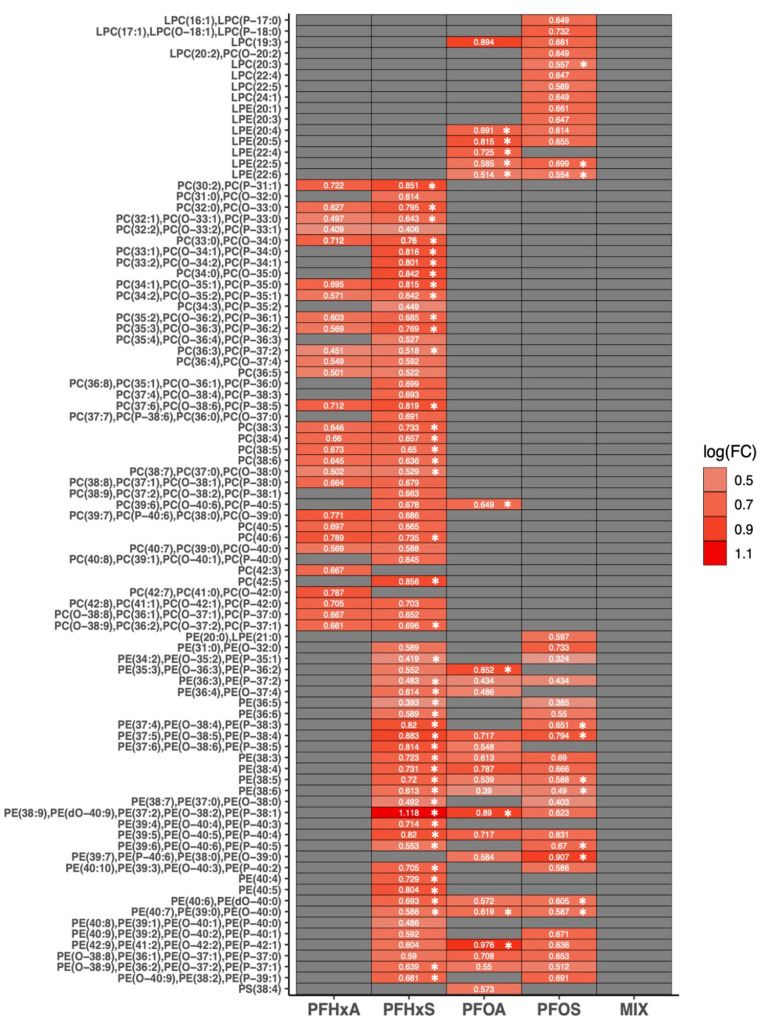
Heat map displaying log2 fold change (logFC) for semi-quantitative ion intensity (relative to internal standards) of hepatic structural lipid species by PFAS treatment, using LIMMA with covariate adjustment (spatial block, developmental stage, genetic sex). Shapes represent FDR-adjusted *p*-values: asterisks, *p* ≤ 0.05; no symbol, *p* ≤ 0.1). Faulty samples identified based on ionization of internal standards and visualization using heatmaps were excluded in analysis. *n* = 21–24 per PFAS treatment (*n* = 5–8 per NF stage/PFAS treatment).

**Figure 4:**
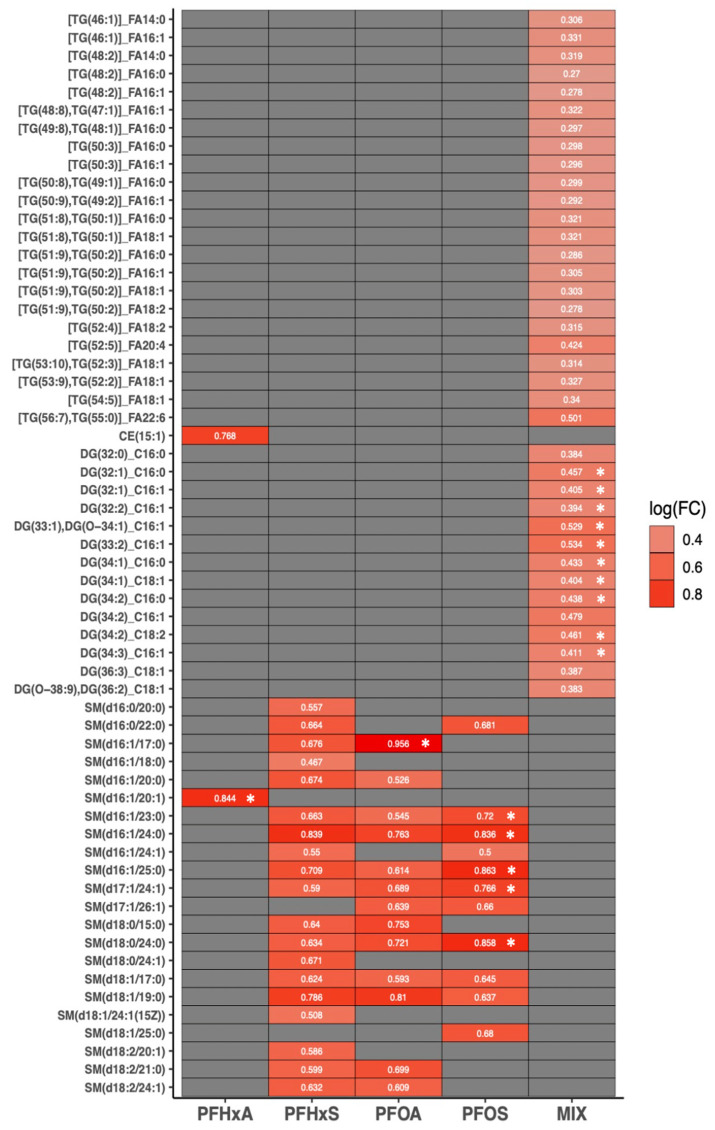
Heat map displaying log2 fold change (logFC) for semi-quantitative ion intensity (relative to internal standards) of hepatic signaling and storage lipid species by PFAS treatment, using LIMMA with covariate adjustment (spatial block, developmental stage, genetic sex). Shapes represent FDR-adjusted *p*-values: asterisks, *p* ≤ 0.05; no symbol, *p* ≤ 0.1). Faulty samples identified based on ionization of internal standards and visualization using heatmaps were excluded in analysis. *n* = 21–24 per PFAS treatment (*n* = 6–8 per NF stage/PFAS treatment).

For our semi-quantitative analysis, we observed significant changes in differential lipids for lysophosphatidylcholines (LPCs), phosphatidylcholines (PCs), PEs, LPEs, diglycerides (DGs), and sphingomyelins (SMs) following exposure to PFAS (Figure 3 and Figure 4; Table S24). Similar to the analysis of relative profiles, we used linear models with covariate adjustment for spatial blocks, NF stage, and genetic sex.

For structural glycerophospholipids, we did not detect significant changes in PI or PG in any treatment, but saw weak evidence (*p* < 0.1) for upregulation of a single PS for PFOA. For lysophospholipids, animals exposed to PFOA or PFOS experienced a significant upregulation in multiple species of LPEs, and for PFOS we observed a significant increase in one LPC species, LPC (20:3). No changes in semiquantitative signatures for lysophospholipids were observed for PFHxA, PFHxS, or our mixture treatment (PFHxS:PFOS). For PFHxS, there was significant, sweeping upregulation in structural phospholipids, specifically PC and PE species, including MRM transitions that include ether lipids (i.e., “O-” and “*P*-”). In contrast, PFHxA had weak evidence for upregulation in PC, but no evidence for changes in PE. For PFOA and PFOS, there was significant upregulation in select PE species and a single PC species for the former (Figure 3; Table S24).

For signaling lipids, we detected no significant changes in CER quantities following PFAS exposure. However, we observed significant upregulation in sphingomyelin (SM) species in PFHxA, PFOA, and PFOS treatments. For PFHxS, we observed weak evidence for SM upregulation (Figure 4; Table S24). For storage lipids, we did not observe significant changes in CE or TG across PFHxA, PFHxS, PFOA, or PFOS, with weak evidence for the upregulation of a single CE in PFHxA. However, we observed a distinct trend in our mixture treatment (PFHxS:PFOS) for the significant upregulation of DG with long chain fatty acids and weak evidence for the upregulation of TG with long chain and very long chain fatty acids.

## 4. Discussion

Growing evidence suggests that PFAS can affect lipid homeostasis, often through interaction with PPARs, in ways that can cause oxidative stress and altered body condition in vertebrates. Here, we exposed African clawed frogs to environmentally relevant levels of PFOS, PFHxS, PFOA, PFHxA, and a mixture of PFOS and PFHxS across larval development and metamorphosis, and found that, despite the absence of effects on apical endpoints (growth, development, and body condition), there were substantial, chemical-specific changes in lipid homeostasis. In contrast with the existing work on adult frogs, we did not detect the expected alterations to storage lipids suggestive of PPAR-mediated energetic disruption. Instead, structural lipids such as glycerophospholipids, SM, and DG were most affected by exposure. When we did observe the PFAS-mediated alteration of liver condition and the expression of genes involved in PPAR signaling, the effects tended to be small and sensitive to both developmental stage and sex. This study adds support to the hypothesis that PFAS alter lipid regulation at environmental levels and that this can occur in the absence of effects on apical measures of body condition associated with lipid regulation. In the context of PFAS-mediated lipid dysregulation, we suggest that developmental exposures might elicit different mechanisms of lipid dysregulation than adult PFAS exposures, and that effects might be mediated by sex and by physiological state, which changes with age and development. Studies that can link lipid dysregulation to fitness consequences are also warranted.

Regardless of the observed molecular effects, we did not see substantial alterations of body condition, nor evidence for non-additive interactions between PFOS and PFHxS for any apical endpoints related to growth or development. Based on the available data, larval stages of *Xenopus laevis* appear robust to apical PFAS effects, with published chronic, larval lowest-observed-effect concentrations (LOECs) for PFOS and PFHxS of 2.5 and 100 mg/L and unbounded no-observed-effect concentrations (NOECs) for PFOA and hexafluoropropylene oxide dimer acid (HFPO-DA or GenX) at 100 mg/L [55]. In contrast, studies with PFOS, PFOA, PFHxS, and 6:2 FTS with North American amphibians, including American bullfrogs (*Rana catesbiana*), Northern leopard frogs, American toads, and Eastern tiger salamanders, report effects at much lower concentrations. Pandelides et al. [23] reviewed 11 available amphibian PFAS toxicity studies and applied conservative criteria for considering effects biologically relevant, including large effect sizes (a 20% difference from control), no deviations from traditional dose responses, no consideration of effects at early time points that later disappear, and SMI was not considered a relevant response. Even under these criteria, chronic LOEC values for PFOS and PFOA were 1.1 mg/L (2.27 times lower than the LOEC for *X*. *laevis*) and 1.4 mg/L (71.43 times lower than the LOEC for *X*. *laevis*). Further, if the risk assessment criteria are relaxed, a very different pattern emerges; LOECs at or below 0.01 mg/L were sometimes observed and LOECs as low as 0.06 ug/L have been reported [23,25]. In contrast to Hoskins et al. [24], effects on SMI during larval stages were not observed in the present study, despite parallel treatments. Altogether, available evidence suggests that *X*. *laevis* is less sensitive than North American anurans to PFOS, PFHxS, and possibly, PFAS, more broadly, although parallel exposures would provide a more definitive test than cross-study comparisons. We hypothesize that this potential difference in species-sensitivity to PFAS is attributable to variation in the abundance and temporal pattern of serum protein concentrations across metamorphosis, which may result in *Xenopus* larvae experiencing a lower body burden relative to other anurans, namely Ranids [33], across development as a result of differences in toxicokinetics at equivalent larval stages [56,57,58]. Regardless of the mechanism, given the popularity of *X*. *laevis* as an amphibian model in ecotoxicology, including its use in multiple assays commonly deployed for regulatory purposes [52,59,60], this apparent difference in sensitivity and mechanisms underlying it should be explored further and considered carefully when interpreting *X*. *laevis* responses for risk assessment purposes.

In contrast to our predictions, we found the observed changes in lipid profiles from PFAS exposure did not align with our expectations for PPAR-mediated alternations, as expression of our selected genes immediately downstream of PPAR*α*, PPAR*β*, and PPAR*γ* was rarely affected. Based on Lin et al. [27] and the frequency with which PPARs have been implicated in PFAS-mediated lipid dysregulation (reviewed by [61]), we expected to see significant changes in the expression of our suite of PPAR-responsive genes, but these changes did not occur. One potential trend for which we observed weak evidence was the downregulation of *apoa5* at NF 62 for *X. laevis* exposed to either PFHxA or PFOA. *Apoa5* has been identified as a direct downstream target of PPARα and encodes for an apolipoprotein that aids in regulating triglyceride homeostasis, with the downregulation of this protein resulting in hypertriglyceridemia in mammals [62]. However, *apoa5* is characteristically upregulated by PPARα agonism through increasing transcription by binding to its promoter region [63]. The weak signal detected is not consistent with PPAR agonism and the underlying mechanism for the observed downregulation remains unclear.

Another unexpected finding was the downregulation of *acox1* for NF 66 males exposed to PFHxS compared to females. Compared to other developmental stages, at NF 66, we had a more balanced number of sexes, and thus increased power to detect significant effects. This downregulation is counterintuitive based on the current literature, which has consistently identified the upregulation of *acox1* as being an indication of PPAR-mediated toxicity in mammalian models and in vitro [64,65,66]. However, Acyl-CoA oxidase 1 produces hydrogen peroxide as a by-product when breaking down fatty acids during β-oxidation, which is a reactive oxygen species (ROS). If exposure to PFHxS is inducing oxidative stress in males through increasing ROS formation, the downregulation of *acox1* could represent a compensatory response to reduce ROS formation associated with fatty acid oxidation and ameliorate cellular oxidative stress [67,68]. At a high exposure concentration, a recent study demonstrated PFHxS impaired glutathione balance in zebrafish embryos and increased ROS almost 4-fold upon exposure [69]. Additionally, our semi-quantitative lipid profiles indicate a sweeping upregulation of glycerophospholipids that may include ether lipids, which have antioxidant properties and may be produced to protect membrane lipids from ROS, or as a consequence of oxidative stress [70,71]. Since peroxisomes are involved in ether lipids synthesis, together this may indicate a peroxisomal response to promote cellular adaptation to low-dose, subchronic PFHxS-induced stress [72].

Although previous PFAS studies with adult amphibians clearly point toward PPAR-mediated lipid dysregulation [27,29,30,31] resulting in altered concentrations of storage lipids, effects on structural and membrane lipids like those observed here are not unprecedented based on the results observed in other taxa. At lower exposure concentrations (0.34 ppb–2.16 ppb), we observed significant treatment-specific changes in glycerophospholipids (LPE, PE, LPC, PC, PI), SM, and DG. Other studies using lipidomic approaches to investigate PFAS effects have also observed changes in these lipid classes, but with variable directionality. In Atlantic cod (*Gadus morhua*), intraperitoneal injections with environmentally representative PFAS mixtures in the ppb dose range (total PFAS, low dose ~52 ppb; high dose ~1030 ppb) elicited an increase in hepatic unsaturated phospholipids (PE, PC, PI), but a decrease in mono-unsaturated TG [73]. In zebrafish embryos exposed to PFHxS (150 ppb, 500 ppb, 1500 ppb, 5000 ppb), an increase in SM along with changes in many species of PC and PE were observed [74]. Notably, at those exposure levels, Xu et al. [74] observed a decrease in ether lipids. However, in chicken embryos injected with PFOS (low dose ~100 ppb; high dose ~1000 ppb), PC, PE, SM, CER, and total TG were all downregulated, although LPE and LPC were upregulated [75]. Interestingly, Kim et al. [76] observed the opposite in *C*. *elegans* exposed to PFOS (500 ppb) and PFOA (2000 ppb) with a decrease in LPE and LPC, and an upregulation in TG by PFOS. These studies highlight the variability in effects on the lipidome following PFAS exposure across various animal models. However, they do support that exposure to PFSA and PFCA may alter the composition of structural and signaling lipids, in addition to storage lipid classes.

A recent review of human epidemiological studies of PFAS effects on the metabolome helps further contextualize the trends we observed across treatments [3]. Across the main PFAS types considered in the review, the legacy compounds PFOS, PFHxS, and PFOA were the most frequently associated with changes in metabolomic profiles, with consistent upregulation in glycerophospholipids (PE, PC) and glycerolipids (TG, DG). The authors noted acyl-carnitines (CARs) and SM were also typically upregulated, but not as consistently. Broadly, our findings in *X*. *laevis* align with these trends, with the exception of limited glycerolipid upregulation for our binary PFSA mixture with the semi-quantitative profile analysis and weak evidence with relative profile analysis for the downregulation in CARs for PFHxS. In the epidemiological literature evaluated, India-Aldana et al. [3] noted metabolites consistently upregulated across studies, four of which had signals detected in this study, either significantly or weakly, for PFHxS and or PFHxA (PC 38:5, PC 38:6, PC 36:5, PC 40:5). Significant changes in PE and PC across PFOS, PFHxS, and PFOA align with epidemiological findings that point to cell membrane disruption, and potentially mitochondrial membrane disruption. In terms of the observed changes for LPE and LPC classes in relative and semi-quantitative profiles, the interpretation for the implications of these classes is somewhat controversial, although biologically relevant, as they play a role in cell–cell signaling to stimulate hepatic cholesterol biosynthesis and decrease fatty acid oxidation [77,78]. Additionally, pointing to membrane disruption, the trends for upregulated sphingomyelins across single-chemical PFAS treatments may indicate changes to membrane fluidity, given their involvement in stabilizing lipid rafts and cholesterol sequestration [79]. Overall, our results for relative and semi-quantitative lipid composition demonstrate PFAS-mediated lipid dysregulation in *X*. *laevis* larvae, predominantly mediated through changes in glycerophospholipids and SM. However, unlike our single-chemical PFSA treatments, there were no detected changes for our binary mixture in the relative or semi-quantitative lipid profiles for glycophospholipids or SM, but a significant increase in DG. Although DG can act as a precursor to both TG and phospholipid synthesis, the weak signal of upregulation of TG species may indicate DG is acting as an intermediate for the former [80].

When viewed in light of the existing studies that exposed adult amphibians to PFAS, observations of disrupted lipid profiles, developmentally transitory (*apoa5, acox1*) and sex-specific effects (*acox1*) on gene expression, and sex-specific effects on SHI in the present study suggest that sex and developmental stage mediate how PFAS influence lipid metabolism in amphibians. For adult male black-spotted frogs, it is clear that PFAS can induce PPAR- and LXR-mediated toxicity [27,29], which we did not observe in our developmental exposures of African clawed frogs. As discussed previously, energetic demands change dramatically across larval development and metamorphosis; the cessation of feeding and massive tissue organization at metamorphosis requires the mobilization of energy reserves acquired during the larval period, and lipid reserves at metamorphosis predict post-metamorphic fitness [20]. In adult amphibians, stored lipid reserves are important for surviving periods of food limitation and are mobilized by both males and females to fuel the energetic demands of reproduction (reviewed by [38]). The focus on adult males by Lin et al. [27,29] was justified by the increased PFAS bioaccumulation in males relative to females, but studies with adult females would be useful for two reasons. On one hand, if the thresholds and severity of effects are positively related to tissue burdens, females might be predicted to respond less severely, given the lower bioaccumulation in the black-spotted frog. On the other hand, oogenesis is a very costly process, so changes to lipid metabolism in females might be predicted to affect reproduction more dramatically than comparable changes in males. Studies with adult *Xenopus* would be useful as a comparison to existing studies with Chinese toads and black-spotted frogs for determining if mechanisms are conserved across disparate amphibian taxa. Altogether, while stage and sex appear to mediate how PFAS influence lipid metabolism and resultant consequences for fitness, more mechanistic studies across life stages are needed, with the ability to test sex differences.

## 5. Conclusions

We have demonstrated stage and sex-specific alterations in hepatic conditions and, notably, PFAS-mediated changes in lipid profiles across larval development and metamorphic climax in *X*. *laevis*. In contrast with previous amphibian work indicating PPAR activation as a primary molecular initiating event, we did not detect overt changes in our selected PPAR-mediated genes, nor in storage lipids. However, due to a lack of mechanistic intervention through the inclusion of either PPAR inhibitors or knockout models, we are limited in our ability to disregard the involvement of PPAR-mediated processes; therefore, future studies should consider the incorporation of such intervention to clarify whether the changes detected in this study may be PPAR-independent. However, the present study does demonstrate changes in structural and membrane-associated lipids in prometamorphic and metamorphosing *X*. *laevis*. Because energetic demands and the associated metabolic processes change dramatically across larval development, metamorphosis, and juvenile and adult stages, during reproductive seasons, and because males and females have been selected to cope with different energetic demands, it follows that both mechanisms and fitness consequences might be predicted to differ with developmental stage and sex. Future investigations of PFAS-mediated lipid dysregulation should explore how ontogeny and sex mitigate mechanisms of toxicity, and even more critically, if and how these changes affect fitness per se. Given that reproductive tests with developmentally exposed amphibians tend to be logistically difficult, field studies that examine relationships between PFAS tissue burdens and fecundity, fertility, reproductive output, and ultimately, population dynamics, would be useful in understanding whether lipid dysregulation can affect wild populations. Ultimately, both an understanding of sex and stage-specific differences in mechanisms and studies aimed at the ascertaining fitness costs associated with these effects are needed.

## Figures and Tables

**Table 1:** Mean measured PFAS concentrations for each treatment from one pooled replicate of exposure water collected on experimental day 3. ND = Not detected. Refer to Table S2 for limits of quantification.

Water Sample	Nominal [µg/L]	PFOS [µg/L]	PFOA [µg/L]	PFHxS [µg/L]	PFHxA [µg/L]	Total PFAS [µg/L]
Control	0	ND	ND	ND	ND	ND
PFOS	0.5	0.666	ND	ND	ND	0.666
PFOA	0.5	0.057	0.503	ND	ND	0.560
PFHxS	0.5	0.065	ND	0.676	ND	0.741
PFHxA	0.5	ND	ND	ND	0.347	0.347
PFOS:PFHxS MIX	0.5:0.5	1.510	ND	0.651	ND	2.16

## Data Availability

Raw data supporting these results can be accessed online through the Purdue University Research Repository (PURR) (https://purr.purdue.edu/publications/4463/1). PDFs of the R outputs for the apical and gene expression analyses can be found with other supplementary documents for this manuscript. Additionally, both data and R code can be access through the following GitHub repository: (https://github.com/bushag/Toxics-Bushong-et-al-2024-10.3390-toxics12100732-.git).

## Supplementary Materials

The following supporting information can be downloaded at: https://www.mdpi.com/article/10.3390/toxics12100732/s1, Figure S1. Marginal mean ± 95% C.I. for (A) wet body mass, (B) snout-vent-length, and (C) Scaled Mass Index (SMI) for X. laevis exposed to different PFAS (Control, Mix (PFHxS:PFOS), PFHxA, PFHxS, PFOA, PFOS) with raw data displayed in the background. Extreme outliers (3 × IQR) excluded. n = 33–36 per *PFAS* treatment (n = 11–12 per NF Stage/PFAS treatment). Figure S2. Results of a Cox proportional hazard analysis for time-to-stage (shown as survival probability on the y-axis). Time-to-stage was not impacted by treatment for NF 58 (A), NF 62 (B), or NF 66 (C). Figure S3. Marginal mean ± 95% C.I. xPPAR target gene (acox1, apoa5, fabp1, pck1) expression in NF 62 livers of X. laevis exposed to different PFAS treatments (Mix (PFHxS:PFOS), PFHxA, PFHxS, PFOA, PFOS). Values relative to NF 62 controls represented by the dashed line at 1.0. Raw data displayed in the background. Extreme outliers (3 × IQR) excluded. n = 6–9 per *PFAS* treatment. Figure S4. Marginal mean ± 95% C.I xPPAR target gene (acox1, apoa5, fabp1, pck1) expression in NF 66 livers of X. laevis exposed to different PFAS treatments (Mix (PFHxS:PFOS), PFHxA, PFHxS, PFOA, PFOS). Values relative to NF 66 controls represented by the dashed line at 1.0. Raw data displayed in the background. Extreme outliers (3 × IQR) excluded. n = 7–9 per *PFAS* treatment. Table S1. Feeding rates adapted from the OECD AMA guidelines for static renewal (OECD, 2009). Table S2. Sample Limit of Quantification (SOQ) for measured PFAS. This measure is not instrumental LOQ and can be considered synonymous with method limit of quantification (MLQ). Table S3. Information on RT-qPCR primers used for real-time quantitative PCR of *X. laevis* xPPARα/β/γ target genes (*apoa5, fabp1, acox1, pck1*) and reference gene (*sub1*). ‘T_A_’ = Annealing Temperature; ‘E%’ = Efficiency calculated from slope of standard curve; ‘[Primer]’ = Concentration of each primer (F & R) per qPCR reaction. Table S4. ANOVA tables (Type III) reporting comparison of mean Mass, SVL, and SMI for *X. laevis* exposed to different PFAS treatments after adjusting for variation in spatial block, sex, and NF Stage. Table S5. ANOVA tables (Type III) reporting comparison of mean Mass, SVL, and SMI for NF58 *X. laevis* exposed to different PFAS treatments after adjusting for variation in spatial block and sex. Table S6. Results of post-hoc Dunnett comparisons of SVL for NF 58 *X. laevis*. Table S7. ANOVA tables (Type III) reporting comparison of mean Mass, SVL, and SMI for NF62 *X. laevis* exposed to different PFAS treatments after adjusting for variation in spatial block and sex. Table S8. Results of post-hoc Dunnett comparisons of SMI for NF 62 *X. laevis*. Table S9. ANOVA tables (Type III) reporting comparison of mean Mass, SVL, and SMI for NF66 *X. laevis* exposed to different PFAS treatments after adjusting for variation in spatial block and sex. Table S10. Results of post-hoc Dunnett comparisons of SMI for NF66 *X. laevis* males by PFAS. Table S11. ANOVA tables (Type III) reporting comparison of mean SHI and HSI for *X. laevis* males and females exposed to different PFAS functional groups after adjusting for variation in spatial block, sex, and NF Stage. Table S12. Results of post-hoc Dunnett comparisons of SHI for female NF 62 *X. laevis* by PFAS. Table S13. Results of post-hoc Dunnett comparisons of SHI for male *X. laevis* by PFAS. Table S14. ANOVA tables (Type III) reporting comparison of mean relative mRNA of *acox1, apoa5, fabp1,* and *pck1* for NF 58 *X. laevis* livers across PFAS treatments after adjusting for variation in spatial block and genetic sex. Table S15. ANOVA tables (Type III) reporting comparison of mean relative mRNA of *acox1, apoa5, fabp1,* and *pck1* for NF 58 *X. laevis* livers across PFAS treatments after adjusting for variation in only spatial block. Table S16. ANOVA tables (Type III) reporting comparison of mean relative mRNA of *apoa5, apoa5, fabp1,* and *pck1* for NF 62 *X. laevis* livers across PFAS treatments after adjusting for variation in spatial block and genetic sex. Table S17. Results of post-hoc Dunnett comparisons of Ln(Fold Change) for *apoa5* and *fabp1* of NF 62 *X. laevis* livers across PFAS treatments after incorporating variation of spatial block and genetic sex. Notably, significant contrast for *fabp1* is likely spurious due to low number of males for that comparison. Table S18. ANOVA tables (Type III) reporting comparison of mean relative mRNA of *apoa5, apoa5, fabp1,* and *pck1* for NF 62 *X. laevis* livers across PFAS treatments after adjusting for variation in only spatial block. Table S19. Results of post-hoc Dunnett comparisons of Ln(Fold Change) for *apoa5* and *fabp1* of NF 62 *X. laevis* livers across PFAS treatments after incorporating variation of only spatial block. Table S20. ANOVA tables (Type III) reporting comparison of mean relative mRNA of *acox1, apoa5, fabp1,* and *pck1* for NF 66 *X. laevis* livers across PFAS treatments after controlling for variation in spatial block and genetic sex. Table S21. Results of post-hoc Dunnett comparisons of Ln(Fold Change) for *acox1* of NF 66 *X. laevis* livers across PFAS treatments for males after incorporating variation of spatial block. Table S22. ANOVA tables (Type III) reporting comparison of mean relative mRNA of *acox1, apoa5, fabp1,* and *pck1* for NF 66 *X. laevis* livers across PFAS treatments after controlling for variation in only spatial block. Table S23. Table reporting pairwise FDR-adjusted contrasts conducted for relative linear models with covariate adjustment for spatial blocks, NF stage, and genetic sex using MetaboAnalyst 5.0. Abbreviation for FDR-adjusted *p*-values are as follows: “*” ≤ 0.05; “.” ≤ 0.1. Table S24. Table reporting pairwise FDR-adjusted contrasts conducted for semi-quantitative linear models with covariate adjustment for spatial blocks, NF stage, and genetic sex using MetaboAnalyst 6.0. Abbreviation for FDR-adjusted *p*-values are as follows: “*” ≤ 0.05; “.” ≤ 0.1.
