# Supplementary material for: Effects of Perfluorinated Alkyl Substances (PFAS) on Amphibian Body and Liver Conditions: Is Lipid Metabolism Being Perturbed throughout Metamorphosis?
Source: Toxics. 2024 Oct 10;12(10):732. doi: 10.3390/toxics12100732 (PMC11510839; doi:10.3390/toxics12100732)
Supplement: Supplementary file 1 [file toxics-12-00732-s001.zip › toxics-3210054-supplementary.pdf]

Table of Contents

Figure S1. Marginal mean ± 95% C.I. for (A) wet body mass, (B) snout-vent-length, and (C) Scaled Mass Index (SMI) for X. laevis exposed to different PFAS (Control, Mix (PFHxS:PFOS), PFHxA, PFHxS, PFOA, PFOS) with raw data displayed in the background. Extreme outliers (3xIQR) excluded. n= 33-36 per *PFAS* treatment (n=11-12 per NF Stage / PFAS treatment).

Figure S2. Results of a Cox proportional hazard analysis for time-to-stage (shown as survival probability on the y-axis). Time-to-stage was not impacted by treatment for NF 58 (A), NF 62 (B), or NF 66 (C).

Figure S3. Marginal mean ± 95% C.I. xPPAR target gene (acox1, apoa5, fabp1, pck1) expression in NF 62 livers of X. laevis exposed to different PFAS treatments (Mix (PFHxS:PFOS), PFHxA, PFHxS, PFOA, PFOS). Values relative to NF 62 controls represented by the dashed line at 1.0. Raw data displayed in the background. Extreme outliers (3 x IQR) excluded. n = 6-9 per *PFAS* treatment.

Figure S4. Marginal mean ± 95% C.I xPPAR target gene (acox1, apoa5, fabp1, pck1) expression in NF 66 livers of X. laevis exposed to different PFAS treatments (Mix (PFHxS:PFOS), PFHxA, PFHxS, PFOA, PFOS). Values relative to NF 66 controls represented by the dashed line at 1.0. Raw data displayed in the background. Extreme outliers (3 x IQR) excluded. n = 7-9 per *PFAS* treatment.

Table S1. Feeding rates adapted from the OECD AMA guidelines for static renewal (OECD, 2009).

Table S2. Sample Limit of Quantification (SOQ) for measured PFAS. This measure is not instrumental LOQ and can be considered synonymous with method limit of quantification (MLQ).

Table S3. Information on RT-qPCR primers used for real-time quantitative PCR of *X. laevis* xPPARα/β/γ target genes (*apoa5, fabp1, acox1, pck1*) and reference gene (*sub1*). 'T_A' = Annealing Temperature; 'E%' = Efficiency calculated from slope of standard curve; '[Primer]' = Concentration of each primer (F & R) per qPCR reaction.

Table S4. ANOVA tables (Type III) reporting comparison of mean Mass, SVL, and SMI for *X. laevis* exposed to different PFAS treatments after adjusting for variation in spatial block, sex, and NF Stage.

Table S5. ANOVA tables (Type III) reporting comparison of mean Mass, SVL, and SMI for NF58 *X. laevis* exposed to different PFAS treatments after adjusting for variation in spatial block and sex.

Table S6. Results of post-hoc Dunnett comparisons of SVL for NF 58 *X. laevis*.

Table S7. ANOVA tables (Type III) reporting comparison of mean Mass, SVL, and SMI for NF62 *X. laevis* exposed to different PFAS treatments after adjusting for variation in spatial block and sex.

Table S8. Results of post-hoc Dunnett comparisons of SMI for NF 62 *X. laevis*.

Table S9. ANOVA tables (Type III) reporting comparison of mean Mass, SVL, and SMI for NF66 *X. laevis* exposed to different PFAS treatments after adjusting for variation in spatial block and sex.

Table S10. Results of post-hoc Dunnett comparisons of SMI for NF66 *X. laevis* males by PFAS.

Table S11. ANOVA tables (Type III) reporting comparison of mean SHI and HSI for *X. laevis* males and females exposed to different PFAS functional groups after adjusting for variation in spatial block, sex, and NF Stage.

Table S12. Results of post-hoc Dunnett comparisons of SHI for female NF 62 *X. laevis* by PFAS.

Table S13. Results of post-hoc Dunnett comparisons of SHI for male *X. laevis* by PFAS.

Table S14. ANOVA tables (Type III) reporting comparison of mean relative mRNA of *acox1, apoa5, fabp1,* and *pck1* for NF 58 *X. laevis* livers across PFAS treatments after adjusting for variation in spatial block and genetic sex.

Table S15. ANOVA tables (Type III) reporting comparison of mean relative mRNA of *acox1, apoa5, fabp1,* and *pck1* for NF 58 *X. laevis* livers across PFAS treatments after adjusting for variation in only spatial block.

Table S16. ANOVA tables (Type III) reporting comparison of mean relative mRNA of *apoa5, apoa5, fabp1,* and *pck1* for NF 62 *X. laevis* livers across PFAS treatments after adjusting for variation in spatial block and genetic sex.

Table S17. Results of post-hoc Dunnett comparisons of Ln(Fold Change) for *apoa5* and *fabp1* of NF 62 *X. laevis* livers across PFAS treatments after incorporating variation of spatial block and genetic sex. Notably, significant contrast for *fabp1* is likely spurious due to low number of males for that comparison.

Table S18. ANOVA tables (Type III) reporting comparison of mean relative mRNA of *apoa5, apoa5, fabp1,* and *pck1* for NF 62 *X. laevis* livers across PFAS treatments after adjusting for variation in only spatial block.

Table S19. Results of post-hoc Dunnett comparisons of Ln(Fold Change) for *apoa5* and *fabp1* of NF 62 *X. laevis* livers across PFAS treatments after incorporating variation of only spatial block.

Table S20. ANOVA tables (Type III) reporting comparison of mean relative mRNA of *acox1, apoa5, fabp1,* and *pck1* for NF 66 *X. laevis* livers across PFAS treatments after controlling for variation in spatial block and genetic sex.

Table S21. Results of post-hoc Dunnett comparisons of Ln(Fold Change) for *acox1* of NF 66 *X. laevis* livers across PFAS treatments for males after incorporating variation of spatial block.

Table S22. ANOVA tables (Type III) reporting comparison of mean relative mRNA of *acox1, apoa5, fabp1,* and *pck1* for NF 66 *X. laevis* livers across PFAS treatments after controlling for variation in only spatial block.

Table S23. Table reporting pairwise FDR-adjusted contrasts conducted for relative linear models with covariate adjustment for spatial blocks, NF stage, and genetic sex using MetaboAnalyst 5.0. Abbreviation for FDR-adjusted p-values are as follows: "*" ≤ 0.05; "." ≤ 0.1.

Table S24. Table reporting pairwise FDR-adjusted contrasts conducted for semi-quantitative linear models with covariate adjustment for spatial blocks, NF stage, and genetic sex using MetaboAnalyst 6.0. Abbreviation for FDR-adjusted p-values are as follows: "*" ≤ 0.05; "." ≤ 0.1.

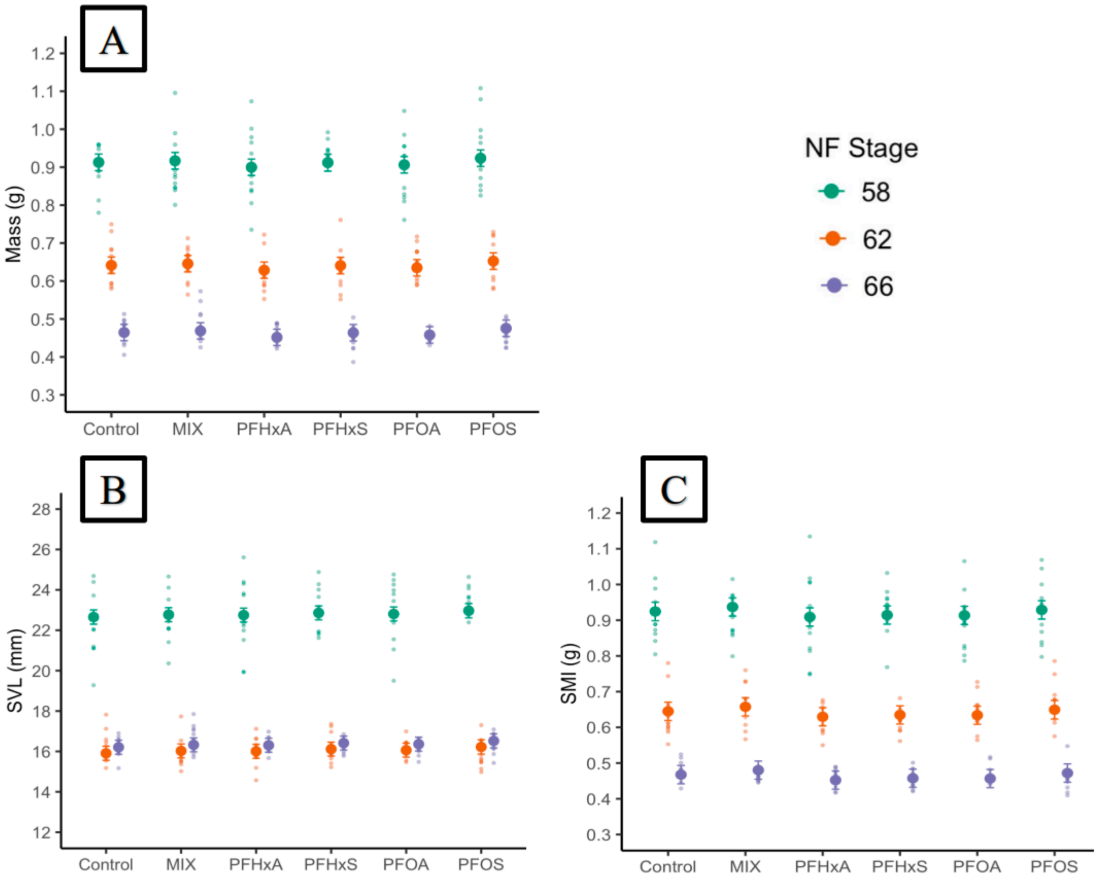

Figure S1. Marginal mean ± 95% C.I. for (A) wet body mass, (B) snout-vent-length, and (C) Scaled Mass Index (SMI) for X. laevis exposed to different PFAS (Control, Mix (PFHxS:PFOS), PFHxA, PFHxS, PFOA, PFOS) with raw data displayed in the background. Extreme outliers (3xIQR) excluded. n= 33-36 per *PFAS* treatment (n=11-12 per NF Stage / PFAS treatment).

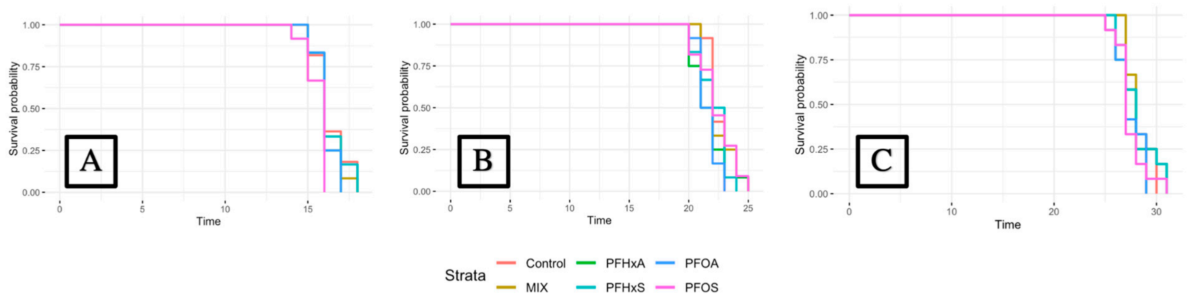

Figure S2. Results of a Cox proportional hazard analysis for time-to-stage (shown as survival probability on the y-axis). Time-to-stage was not impacted by treatment for NF 58 (A), NF 62 (B), or NF 66 (C).

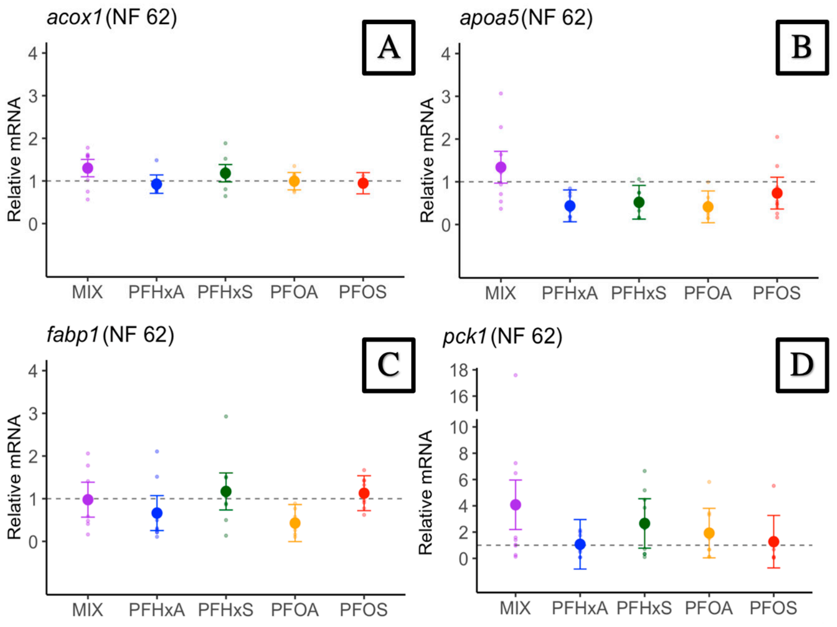

Figure S3. Marginal mean ± 95% C.I. xPPAR target gene (acox1, apoa5, fabp1, pck1) expression in NF 62 livers of X. laevis exposed to different PFAS treatments (Mix (PFHxS:PFOS), PFHxA, PFHxS, PFOA, PFOS). Values relative to NF 62 controls represented by the dashed line at 1.0. Raw data displayed in the background. Extreme outliers (3 x IQR) excluded. n = 6-9 per *PFAS* treatment.

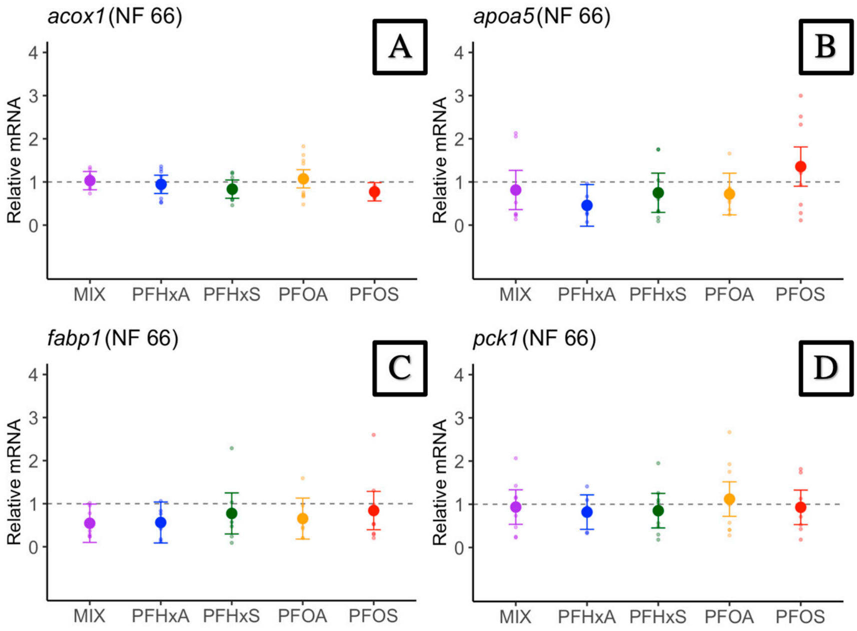

Figure S4. Marginal mean ± 95% C.I xPPAR target gene (acox1, apoa5, fabp1, pck1) expression in NF 66 livers of X. laevis exposed to different PFAS treatments (Mix (PFHxS:PFOS), PFHxA, PFHxS, PFOA, PFOS). Values relative to NF 66 controls represented by the dashed line at 1.0. Raw data displayed in the background. Extreme outliers (3 x IQR) excluded. n = 7-9 per *PFAS* treatment.

Table S1. Feeding rates adapted from the OECD AMA guidelines for static renewal (OECD, 2009).

**Days Post-Fertilization**	**Food Ration (mg of Sera Micron per animal/day)**
4-8	5
9-12	9
13-17	13
18-22	15
23-25	18
26-28	20
29-32	25
33-39	28

Table S2. Sample Limit of Quantification (SOQ) for measured PFAS. This measure is not instrumental LOQ and can be considered synonymous with method limit of quantification (MLQ).

Table S3. Information on RT-qPCR primers used for real-time quantitative PCR of *X. laevis* xPPAR$\alpha/\beta/\gamma$ target genes (*apoa5, fabp1, acox1, pck1*) and reference gene (*sub1*). 'T_A' = Annealing Temperature; 'E%' = Efficiency calculated from slope of standard curve; '[Primer]' = Concentration of each primer (F & R) per qPCR reaction.

Compound	PFOS [ug/L]	PFOA [ug/L]	PFHxS [ug/L]	PFHxA [ug/L]
**LOQ [ug/L]**	0.042	0.059	0.091	0.012

Target Gene	F/Left Primer (5′-3′)	R/Right Primer (5′-3′)	T_A (°C)	E%	[Primer]	Amplicon	Spans Exon?	NCBI	Source	DOI
acox1.L	ACATGGGATCAGCAAGAACAC	CCACTGCATTAGGACGGATTA	51.9	92.1	250 nM	127 bp	Yes	NM_001096680.1	Tamaoki et al., 2018	https://doi.org/10.1002/jez.2246
apoa5.L	CCGGAAGAGTTAAGGAAGGTTT	GCTCAGCTTATCCTGTGTTGTG	51.9	90.5	250 nM	125 bp	No	NM_001110744.1	Self-designed	NA
fabp1	TAAAGGGTGTCACCGAGATTG	TCTCCCCTGTTGGTGTTTCTA	51.9	92.3	188 nM	129 bp	No	AF068301.1	Tamaoki et al., 2018	https://doi.org/10.1002/jez.2246
pck1.S	GCAGCTGAACATAAAGGCAAG	TGAGCCAGTGAGCAAGGTATT	53	92.7	250 nM	97 bp	Yes	NM_001086477.1	Tamaoki et al., 2018	https://doi.org/10.1002/jez.2246
sub1.L	AGCAGGAGAAATGAAGCCAGG	CCGACATCTGCTCCTTCAGT	53.5	97.3	188 nM	80 bp	Yes	XM_018266540.2	Mughal et al., 2018	https://doi.org/10.1038/s41598-017-18684-1

Table S4. ANOVA tables (Type III) reporting comparison of mean Mass, SVL, and SMI for *X. laevis* exposed to different PFAS treatments after adjusting for variation in spatial block, sex, and NF Stage. Notably, these models had issues of heteroskedasticity, which supported our decision to proceed with analyzing by NF stage.

.

Endpoint	Source of Variance	SS	df	*F*	*p*	Sig
**Mass**	(Intercept)	8.914	1	2771.456	0.000	***
	Block	0.008	2	1.188	0.308	ns

	Sex	0.000	1	0.006	0.939	ns
	NF Stage	0.643	2	100.004	0.000	***
	PFAS	0.032	5	1.976	0.085	.
	Sex:NF Stage	0.007	2	1.124	0.328	ns
	Sex:PFAS	0.009	5	0.562	0.729	ns
	NF Stage:Treatment	0.045	10	1.406	0.182	ns
	Sex:NF Stage:PFAS	0.027	10	0.826	0.605	ns
	Residuals	0.544	169			
**SVL**	(Intercept)	6426.059	1	8295.971	0.000	***
	Block	1.625	2	1.049	0.353	ns
	Sex	0.826	1	1.067	0.303	ns
	NF Stage	134.829	2	87.031	0.000	***
	PFAS	4.866	5	1.256	0.285	ns
	Sex:NF Stage	9.008	2	5.815	0.004	**
	Sex:PFAS	3.219	5	0.831	0.529	ns
	NF Stage:PFAS	20.813	10	2.687	0.004	**
	Sex:NF Stage:PFAS	17.667	10	2.281	0.016	*
	Residuals	134.006	173			
**SMI**	(Intercept)	8.804	1	2547.495	0.000	***
	Block	0.001	2	0.087	0.916	ns
	Sex	0.000	1	0.019	0.890	ns
	NF Stage	0.788	2	113.993	0.000	***
	PFAS	0.020	5	1.168	0.327	ns
	Sex:NF Stage	0.007	2	1.034	0.358	ns
	Sex:PFAS	0.019	5	1.117	0.353	ns
	NF Stage:PFAS	0.034	10	0.997	0.448	ns
	Sex:NF Stage:PFAS	0.023	10	0.676	0.746	ns
	Residuals	0.587	170			

Table S5. ANOVA tables (Type III) reporting comparison of mean Mass, SVL, and SMI for NF58 *X. laevis* exposed to different PFAS treatments after adjusting for variation in spatial block and sex.

**Endpoint**	**Source of Variance**	**SS**	**df**	***F***	***p***	**Sig**
**Mass** (NF 58)	(Intercept)	4.671	1	716.560	0.000	***
	Block	0.000	2	0.032	0.968	ns

	Sex	0.004	1	0.632	0.430	ns
	PFAS	0.050	5	1.528	0.197	ns
	Sex:PFAS	0.030	5	0.919	0.476	ns
	Residuals	0.339	52			
**SVL** (NF 58)	(Intercept)	2775.302	1	1583.623	0.000	***
	Block	4.143	2	1.182	0.314	ns
	Sex	7.337	1	4.187	0.046	*
	PFAS	17.425	5	1.989	0.095	.
	Sex:PFAS	12.636	5	1.442	0.224	ns
	Residuals	94.635	54			
**SVL** (males) (NF 58)	(Intercept)	1508.057	1	1354.002	0.000	***
	Block	5.515	2	2.476	0.107	ns
	PFAS	3.4800	5	0.625	0.682	ns
	Residuals	24.503	22			
**SVL** (females) (NF 58)	(Intercept)	2521.602	1	1107.768	0.000	***
	Block	0.471	2	0.104	0.902	ns
	PFAS	18.650	5	1.639	0.180	ns
	Residuals	68.289	30			
**SMI** (NF 58)	(Intercept)	4.999	1	679.805	0.000	***
	Block	0.013	2	0.851	0.432	ns
	Sex	0.001	1	0.191	0.664	ns
	PFAS	0.022	5	0.587	0.710	ns
	Sex:PFAS	0.008	5	0.208	0.958	ns
	Residuals	0.397	54			

Table S6. Results of post-hoc Dunnett comparisons of SVL for NF 58 *X. laevis*.

**Endpoint**	**Comparison**	**Estimates**	**SE**	**t-value**	***p***	**Sig**
**SVL**	PFHxA-Control	0.394	0.710	0.555	0.975	ns
	PFOA-Control	1.324	0.676	1.957	0.204	ns
	PFHxS-Control	1.435	0.710	2.020	0.181	ns
	PFOS-Control	2.148	0.840	2.558	0.055	.

MIX-Control	0.477	0.832	0.574	0.972	ns

Table S7. ANOVA tables (Type III) reporting comparison of mean Mass, SVL, and SMI for NF62 *X. laevis* exposed to different PFAS treatments after adjusting for variation in spatial block and sex.

Endpoint	Source of Variance	SS	df	*F*	*p*	Sig
**Mass** (NF 62)	(Intercept)	3.295	1	1262.625	0.000	***
	Block	0.008	2	1.505	0.231	ns
	Sex	0.003	1	0.974	0.328	ns
	PFAS	0.012	5	0.897	0.489	ns
	Sex:PFAS	0.005	5	0.406	0.843	ns
	Residuals	0.146	56			
**SVL** (NF 62)	(Intercept)	2014.863	1	5156.715	0.000	***
	Block	2.666	2	3.412	0.040	*
	Sex	0.225	1	0.575	0.452	ns
	PFAS	2.849	5	1.458	0.218	ns
	Sex:PFAS	2.768	5	1.417	0.232	ns
	Residuals	22.271	57			
**SMI** (NF 62)	(Intercept)	3.061	1	1278.534	0.000	***
	Block	0.013	2	2.781	0.070	.
	Sex	0.000	1	0.047	0.828	ns
	PFAS	0.038	5	3.179	0.013	*
	Sex:PFAS	0.027	5	2.220	0.065	.
	Residuals	0.136	57			
**SMI** (males) (NF 62)	(Intercept)	0.703	1	299.082	0.000	***
	Block	0.011	2	2.388	0.120	ns
	PFAS	0.011	5	0.958	0.469	ns
	Residuals	0.042	18			
**SMI** (females) (NF 62)	(Intercept)	2.833	1	1155.947	0.000	***
	Block	0.006	2	1.139	0.331	ns
	PFAS	0.038	5	3.088	0.020	*
	Residuals	0.091	37			

Table S8. Results of post-hoc Dunnett comparisons of SMI for NF 62 *X. laevis*.

Endpoint	Comparison	Estimates	SE	t-value	*p*	Sig
**SMI** (females)	PFHxA-Control	-0.025	0.025	-0.991	0.797	ns
	PFOA-Control	-0.013	0.023	-0.574	0.972	ns
	PFHxS-Control	-0.011	0.031	-0.359	0.997	ns
	PFOS-Control	0.047	0.024	1.915	0.23	ns
	MIX-Control	0.048	0.025	1.921	0.228	ns

Table S9. ANOVA tables (Type III) reporting comparison of mean Mass, SVL, and SMI for NF66 *X. laevis* exposed to different PFAS treatments after adjusting for variation in spatial block and sex.

Endpoint	Source of Variance	SS	df	*F*	*p*	Sig
**Mass** (NF 66)	(Intercept)	1.397	1	1523.000	0.000	***
	Block	0.006	2	3.024	0.056	.
	Sex	0.000	1	0.009	0.926	ns
	PFAS	0.005	5	1.089	0.376	ns
	Sex:PFAS	0.001	5	0.240	0.943	ns
	Residuals	0.052	57			
**SVL** (NF 66)	(Intercept)	1687.827	1	9014.553	0.000	***
	Block	1.055	2	2.818	0.068	.
	Sex	0.771	1	4.118	0.047	*
	PFAS	1.067	5	1.140	0.350	ns
	Sex:PFAS	0.894	5	0.955	0.453	ns
	Residuals	10.860	58			
**SVL** (males) (NF 66)	(Intercept)	888.152	1	4219.117	0.000	***
	Block	0.581	2	1.380	0.271	ns
	PFAS	1.319	5	1.253	0.316	ns
	Residuals	5.052	24			
**SVL** (females) (NF 66)	(Intercept)	1427.384	1	7980.12	0.000	***
	Block	0.558	2	1.560	0.226	ns
	PFAS	0.952	5	1.065	0.398	ns
	Residuals	5.724	32			
**SMI** (NF 66)	(Intercept)	1.270	1	2481.580	0.000	***
	Block	0.001	2	0.550	0.580	ns

	Sex	0.004	1	7.516	0.008	**
	PFAS	0.002	5	0.633	0.675	ns
	Sex:PFAS	0.003	5	1.014	0.418	ns
	Residuals	0.028	55			
**SMI** (males) (NF 66)	(Intercept)	0.837	1	1312.434	0.000	***
	Block	0.001	2	0.882	0.427	ns
	PFAS	0.007	5	2.159	0.093	.
	Residuals	0.015	24			
**SMI** (females) (NF 66)	(Intercept)	1.073	1	2596.635	0.000	***
	Block	0.000	2	0.358	0.702	ns
	PFAS	0.001	5	0.725	0.610	ns
	Residuals	0.012	29			

Table S10. Results of post-hoc Dunnett comparisons of SMI for NF66 *X. laevis* males by PFAS.

**Endpoint**	**Comparison**	**Estimates**	**SE**	**t-value**	***p***	**Sig**
**SMI** (males)	PFHxA-Control	-0.042	0.018	-2.337	0.098	.
	PFOA-Control	-0.026	0.017	-1.518	0.405	ns
	PFHxS-Control	-0.038	0.016	-2.390	0.089	.
	PFOS-Control	-0.055	0.018	-3.053	0.021	*
	MIX-Control	-0.034	0.016	-2.172	0.136	ns

Table S11. ANOVA tables (Type III) reporting comparison of mean SHI and HSI for *X. laevis* males and females exposed to different PFAS after adjusting for variation in spatial block, sex, and NF Stage.

**Endpoint**	**Source of Variance**	**SS**	**df**	***F***	***p***	**Sig**
**SHI**	(Intercept)	1005.223	1	604.848	0.000	ns
	Block	1.884	2	0.567	0.568	ns
	Sex	2.419	1	1.456	0.229	ns
	NF Stage	77.414	2	23.290	0.000	***
	PFAS	13.576	5	1.634	0.154	ns
	Sex:NF Stage	5.067	2	1.524	0.221	ns
	Sex:PFAS	20.547	5	2.473	0.034	*

	NF Stage:PFAS	34.105	10	2.052	0.031	*
	Sex:NF Stage:PFAS	33.612	10	2.022	0.034	*
	Residuals	282.530	170			
**SHI** (males)	(Intercept)	477.104	1	357.092	0.000	***
	Block	0.330	2	0.124	0.884	ns
	NF Stage	22.440	2	8.398	0.001	**
	PFAS	16.768	5	2.510	0.039	*
	NF Stage:PFAS	14.027	10	1.050	0.413	ns
	Residuals	86.845	65			
**SHI** (females)	(Intercept)	897.366	1	473.454	0.000	***
	Block	2.017	2	0.532	0.589	ns
	NF Stage	76.981	2	20.308	0.000	***
	PFAS	13.717	5	1.447	0.214	ns
	NF Stage:PFAS	34.048	10	1.796	0.070	.
	Residuals	195.222	103			
**SHI** (females) (NF 62)	(Intercept)	541.49	1	205.173	0.000	***
	Block	2.172	2	0.412	0.666	ns
	PFAS	30.853	5	2.338	0.061	.
	Residuals	97.65	37			
**HSI**	(Intercept)	24.305	1	1235.344	0.000	***
	Block	0.063	2	1.597	0.206	ns
	Sex	0.031	1	1.566	0.212	ns
	NF Stage	1.357	2	34.497	0.000	***
	PFAS	0.183	5	1.858	0.104	ns
	Sex:NF Stage	0.003	2	0.074	0.928	ns
	Sex:PFAS	0.154	5	1.567	0.172	ns
	NF Stage:PFAS	0.195	10	0.990	0.454	ns
	Sex:NF Stage:PFAS	0.258	10	1.313	0.227	ns
	Residuals	3.384	172			
**HSI** (males)	(Intercept)	11.669	1	504.315	0.000	***
	Block	0.049	2	1.051	0.355	ns
	NF Stage	0.691	2	14.937	0.000	***
	PFAS	0.174	5	1.504	0.200	ns

	NF Stage:PFAS	0.178	10	0.768	0.659	ns
	Residuals	1.597	69			
**HSI** (females)	(Intercept)	20.975	1	1206.468	0.000	***
	Block	0.046	2	1.311	0.274	ns
	NF Stage	1.353	2	38.903	0.000	***
	PFAS	0.192	5	2.209	0.059	.
	NF Stage:PFAS	0.204	10	1.173	0.318	ns
	Residuals	1.756	101			

Table S12. Results of post-hoc Dunnett comparisons of SHI for female NF 62 *X. laevis* by PFAS.

**Endpoint**	**Comparison**	**Estimates**	**SE**	**t-value**	***p***	**Sig**
**SHI** (females)						
(NF 62)	PFHxA-Control	-0.294	0.814	-0.362	0.996	ns
	PFOA-Control	-0.341	0.766	-0.445	0.991	ns
	PFHxS-Control	-0.631	1.013	-0.623	0.961	ns
	PFOS-Control	1.679	0.798	2.104	0.161	ns
	MIX-Control	1.138	0.824	1.381	0.531	ns

Table S13. Results of post-hoc Dunnett comparisons of SHI for male *X. laevis* by PFAS.

**Endpoint**	**Comparison**	**Estimates**	**SE**	**t-value**	***p***	**Sig**
**SHI** (males)	PFHxA-Control	0.034	0.487	0.070	1	ns
	PFOA-Control	-0.139	0.505	-0.276	0.999	ns
	PFHxS-Control	-0.483	0.453	-1.068	0.706	ns
	PFOS-Control	-1.311	0.485	-2.703	0.035	*
	MIX-Control	-0.773	0.451	-1.712	0.290	ns

Table S14. ANOVA tables (Type III) reporting comparison of mean relative mRNA of *acox1, apoa5, fabp1,* and *pck1* for NF 58 *X. laevis* livers across PFAS treatments after adjusting for variation in spatial block and genetic sex.

**Target Gene**	**Source of Variance**	**SS**	**df**	***F***	***p***	**Sig**

acox1 (NF 58)	(Intercept)	0.021	1	0.187	0.668	ns
	Block	0.058	2	0.255	0.776	ns
	Sex	0.028	1	0.243	0.625	ns
	PFAS	0.228	5	0.403	0.843	ns
	Sex:PFAS	0.505	5	0.892	0.497	ns
	Residuals	4.077	36			
apoa5 (NF 58)	(Intercept)	0.107	1	0.117	0.735	ns
	Block	1.238	2	0.673	0.517	ns
	Sex	0.901	1	0.979	0.329	ns
	PFAS	1.539	5	0.335	0.889	ns
	Sex:PFAS	2.513	5	0.546	0.740	ns
	Residuals	33.116	36			
fabp1 (NF 58)	(Intercept)	0.105	1	0.179	0.675	ns
	Block	0.232	2	0.196	0.823	ns
	Sex	0.032	1	0.054	0.817	ns
	PFAS	1.743	5	0.590	0.707	ns
	Sex:PFAS	3.342	5	1.132	0.361	ns
	Residuals	21.853	37			
pck1 (NF 58)	(Intercept)	1.266	1	1.130	0.296	ns
	Block	2.767	2	1.234	0.303	ns
	Sex	0.567	1	0.505	0.482	ns
	PFAS	5.954	5	1.062	0.398	ns
	Sex:PFAS	3.199	5	0.571	0.722	ns
	Residuals	39.234	35			

Table S15. ANOVA tables (Type III) reporting comparison of mean relative mRNA of *acox1, apoa5, fabp1,* and *pck1* for NF 58 *X. laevis* livers across PFAS treatments after adjusting for variation in only spatial block.

**Target Gene**	**Source of Variance**	**SS**	**df**	***F***	***p***	**Sig**
acox1 (NF 58)	(Intercept)	0.044	1	0.408	0.526	ns
	Block	0.217	2	0.996	0.378	ns
	PFAS	0.920	5	1.689	0.157	ns
	Residuals	4.764	44			
apoa5 (NF 58)	(Intercept)	1.203	1	1.473	0.231	ns

	Block	0.976	1	0.598	0.554	ns
	PFAS	6.212	5	1.522	0.203	ns
	Residuals	36.921	44			
fabp1 (NF 58)	(Intercept)	0.082	1	0.138	0.712	ns
	Block	0.360	2	0.302	0.741	ns
	PFAS	2.255	5	0.756	0.587	ns
	Residuals	26.867	45			
pck1 (NF 58)	(Intercept)	0.285	1	0.272	0.605	ns
	Block	1.777	2	0.846	0.436	ns
	PFAS	10.559	5	2.012	0.096	.
	Residuals	45.140	43			

Table S16. ANOVA tables (Type III) reporting comparison of mean relative mRNA of *apoa5, apoa5, fabp1,* and *pck1* for NF 62 *X. laevis* livers across PFAS treatments after adjusting for variation in spatial block and genetic sex.

Target Gene	Source of Variance	SS	df	*F*	*p*	Sig
acox1 (NF 62)	(Intercept)	0.050	1	0.557	0.460	ns
	Block	0.226	2	1.263	0.295	ns
	Sex	0.000	1	0.005	0.945	ns
	PFAS	0.248	5	0.554	0.735	ns
	Sex:PFAS	0.058	5	0.130	0.984	ns
	Residuals	3.226	36			
apoa5 (NF 62)	(Intercept)	0.190	1	0.440	0.511	ns
	Block	2.260	2	2.618	0.086	.
	Sex	0.410	1	0.951	0.336	ns
	PFAS	10.580	5	4.901	0.001	***
	Sex:PFAS	4.183	5	1.938	0.110	ns
	Residuals	16.837	39			
fabp1 (NF 62)	(Intercept)	0.016	1	0.025	0.876	ns
	Block	0.598	2	0.462	0.633	ns
	Sex	1.703	1	2.633	0.113	ns
	PFAS	8.486	5	2.623	0.040	*
	Sex:PFAS	2.572	5	0.795	0.560	ns

	Residuals	23.941	37			
pck1 (NF 62)	(Intercept)	0.173	1	0.087	0.770	ns
	Block	0.358	2	0.090	0.914	ns
	Sex	0.148	1	0.074	0.787	ns
	PFAS	20.068	5	2.017	0.098	.
	Sex:PFAS	14.807	5	1.488	0.216	ns
	Residuals	77.623	39			

Table S17. Results of post-hoc Dunnett comparisons of Ln(Fold Change) for *apoa5* and *fabp1* of NF 62 *X. laevis* livers across PFAS treatments after incorporating variation of spatial block and genetic sex. Notably, significant contrast for PFOA for *fabp1* and weak signal for *apoa5* is likely spurious due to low number of males for that comparison.

Target Gene	Comparison	Estimates	SE	t-value	*p*	Sig
apoa5 (NF 62)	MIX-Control	0.697	0.391	1.784	0.288	ns
	PFHxA-Control	-0.938	0.370	-2.533	0.064	.
	PFHxS-Control	-0.174	0.464	-0.374	0.996	ns
	PFOA-Control	-0.880	0.341	-2.584	0.057	.
	PFOS-Control	-0.300	0.353	-0.841	0.875	ns
fabp1 (NF 62)	MIX-Control	-0.389	0.502	-0.775	0.905	ns
	PFHxA-Control	-1.014	0.472	-2.146	0.145	ns
	PFHxS-Control	-0.104	0.695	-0.150	1.000	ns
	PFOA-Control	-1.219	0.454	-2.685	0.044	*
	PFOS-Control	-0.038	0.454	-0.083	1.000	ns

Table S18. ANOVA tables (Type III) reporting comparison of mean relative mRNA of *apoa5, apoa5, fabp1,* and *pck1* for NF 62 *X. laevis* livers across PFAS treatments after adjusting for variation in only spatial block.

Target Gene	Source of Variance	SS	df	*F*	*p*	Sig
acox1 (NF 62)	(Intercept)	0.049	1	0.631	0.431	ns
	Block	0.220	2	1.401	0.258	ns
	PFAS	0.628	5	1.603	0.180	ns
	Residuals	3.291	42			

apoa5 (NF 62)	(Intercept)	0.102	1	0.214	0.646	ns
	Block	3.380	2	3.555	0.037	*
	PFAS	9.947	5	4.185	0.003	**
	Residuals	22.390	45			
fabp1 (NF 62)	(Intercept)	0.448	1	0.726	0.399	ns
	Block	0.845	2	0.685	0.509	ns
	PFAS	8.456	5	2.743	0.031	*
	Residuals	26.515	43			
pck1 (NF 62)	(Intercept)	0.054	1	0.026	0.872	ns
	Block	0.771	2	0.188	0.830	ns
	PFAS	8.769	5	0.854	0.519	ns
	Residuals	92.433	45			

Table S19. Results of post-hoc Dunnett comparisons of Ln(Fold Change) for *apoa5* and *fabp1* of NF 62 *X. laevis* livers across PFAS treatments after incorporating variation of only spatial block.

**Target Gene**	**Comparison**	**Estimates**	**SE**	**t-value**	***p***	**Sig**
apoa5 (NF 62)	MIX-Control	0.330	0.325	1.014	0.767	ns
	PFHxA-Control	-0.811	0.325	-2.495	0.065	.
	PFHxS-Control	-0.639	0.335	-1.904	0.222	ns
	PFOA-Control	-0.840	0.325	-2.585	0.053	.
	PFOS-Control	-0.355	0.325	-1.093	0.714	ns
fabp1 (NF 62)	MIX-Control	-0.036	0.382	-0.095	1.000	ns
	PFHxA-Control	-0.613	0.382	-1.605	0.363	ns
	PFHxS-Control	0.125	0.394	0.318	0.998	ns
	PFOA-Control	-0.836	0.394	-2.122	0.143	ns
	PFOS-Control	0.311	0.382	0.813	0.878	ns

Table S20. ANOVA tables (Type III) reporting comparison of mean relative mRNA of *acox1, apoa5, fabp1,* and *pck1* for NF 66 *X. laevis* livers across PFAS treatments after controlling for variation in spatial block and genetic sex.

**Target Gene**	**Source of Variance**	**SS**	**df**	***F***	***p***	**Sig**

acox1 (NF 66)	(Intercept)	0.131	1	1.320	0.257	ns
	Block	0.183	2	0.926	0.405	ns
	Sex	0.131	1	1.321	0.257	ns
	PFAS	0.375	5	0.756	0.587	ns
	Sex:PFAS	1.174	5	2.369	0.057	.
	Residuals	3.964	40			
acox1 (males) (NF 66)	(Intercept)	0.049	1	1.446	0.248	ns
	Block	0.037	2	0.548	0.589	ns
	PFAS	1.471	5	8.705	<0.001	***
	Residuals	0.507	15			
acox1 (females) (NF 66)	(Intercept)	0.200	1	1.629	0.215	ns
	Block	0.279	2	1.133	0.340	ns
	PFAS	0.375	5	0.611	0.693	ns
	Residuals	2.705	22			
apoa5 (NF 66)	(Intercept)	0.819	1	1.297	0.262	ns
	Block	12.751	2	10.09	<0.001	***
	Sex	0.037	1	0.059	0.809	ns
	PFAS	2.380	5	0.754	0.589	ns
	Sex:PFAS	0.724	5	0.229	0.948	ns
	Residuals	24.008	38			
fabp1 (NF 66)	(Intercept)	0.157	1	0.263	0.611	ns
	Block	3.430	2	2.870	0.072	.
	Sex	0.213	1	0.357	0.555	ns
	PFAS	3.429	5	1.147	0.358	ns
	Sex:PFAS	5.441	5	1.821	0.139	ns
	Residuals	17.929	30			
pck1 (NF 66)	(Intercept)	0.413	1	0.701	0.408	ns
	Block	1.380	2	1.171	0.320	ns
	Sex	0.222	1	0.376	0.543	ns
	PFAS	0.722	5	0.245	0.940	ns
	PFAS:Sex	1.401	5	0.475	0.792	ns
	Residuals	23.570	40			

Table S21. Results of post-hoc Dunnett comparisons of Ln(Fold Change) for *acox1* of NF 66 *X. laevis* livers across PFAS treatments for males after incorporating variation of spatial block.

Target Gene	Comparison	Estimates	SE	t-value	*p*	Sig
acox1 (males) (NF 66)	MIX-Control	0.025	0.139	0.181	1.000	ns
	PFHxA-Control	0.087	0.154	0.568	0.961	ns
	PFHxS-Control	-0.543	0.139	-3.909	0.005	**
	PFOA-Control	-0.442	0.169	-2.681	0.061	.
	PFOS-Control	-0.352	0.148	-2.374	0.107	ns

Table S22. ANOVA tables (Type III) reporting comparison of mean relative mRNA of *acox1, apoa5, fabp1,* and *pck1* for NF 66 *X. laevis* livers across PFAS treatments after controlling for variation in only spatial block.

Target Gene	Source of Variance	SS	df	*F*	*p*	Sig
acox1 (NF 66)	(Intercept)	0.049	1	0.442	0.509	ns
	Block	0.206	2	0.923	0.405	ns
	PFAS	0.597	5	1.069	0.390	ns
	Residuals	5.139	46			
apoa5 (NF 66)	(Intercept)	0.904	1	01.569	0.217	ns
	Block	13.836	2	12.011	<0.001	ns
	PFAS	4.045	5	1.405	0.241	ns
	Residuals	25.343	44			
fabp1 (NF 66)	(Intercept)	0.000	1	0.000	0.999	ns
	Block	2.541	2	1.940	0.158	.
	PFAS	0.862	5	0.263	0.930	ns
	Residuals	23.579	36			
pck1 (NF 66)	(Intercept)	0.185	1	0.338	0.564	ns
	Block	1.095	2	1.003	0.375	ns
	PFAS	0.248	5	0.091	0.993	ns
	Residuals	25.111	46			

Table S23. Table reporting pairwise FDR-adjusted contrasts conducted for relative linear models with covariate adjustment for spatial blocks, NF stage, and genetic sex using MetaboAnalyst 5.0. Abbreviation for FDR-adjusted p-values are as follows: "*" ≤ 0.05; "." ≤ 0.1.

Treatment	Lipid Class	Tentative Lipid ID	Log(FC)	t-value	*p*	*adj p*	Sig
**PFHxA**	Lysophosphatidyl-ethanolamines	LPE(18:1)	-0.654	-2.4168	0.016	0.065	.
		LPE(22:4)	-0.727	-2.7799	0.006	0.064	.
		LPE(24:6)	-0.668	-2.5707	0.011	0.064	.
	Phosphatidyl-cholines	PC(43:4),PC(O-44:4)	-0.829	-3.5066	<0.001	0.068	.
	Phospho-ethanolamines	PE(35:4),PE(O-36:4),PE(P-36:3)	-0.901	-3.4394	<0.001	0.039	*
**PFHxS**	Acyl carnitines	CAR	-0.542	-1.8766	0.061	0.098	.
		CAR_QUAL	-0.597	-2.067	0.039	0.094	.
		CAR(17:0)	-0.87	-3.0142	0.003	0.045	.
		CAR(17:0)_QUAL	-0.501	-1.7336	0.083	0.115	
		CAR(2:0)	-0.538	-1.8644	0.062	0.098	.
		CAR(2:0)_QUAL	-0.552	-1.9135	0.056	0.098	.
		CAR(20:0)	-0.722	-2.5013	0.012	0.062	.
		CAR(20:0)_QUAL	-0.561	-1.9447	0.052	0.098	.
		CAR(22:0)	-0.738	-2.5555	0.011	0.062	.
		CAR(22:0)_QUAL	-0.558	-1.932	0.053	0.098	.
		CAR(22:2)	-0.626	-2.1674	0.030	0.083	.
		CAR(22:2)_QUAL	-0.567	-1.9632	0.050	0.098	.
		CAR(22:4)	-0.597	-2.067	0.039	0.094	.
		CAR(22:4)_QUAL	-0.512	-1.7731	0.076	0.114	
	Lysophosphatidyl-ethanolamines	LPE(16:1)	-0.603	-2.2446	0.025	0.061	.
		LPE(18:1)	-0.554	-2.0299	0.043	0.065	.
		LPE(18:2),LPE(P-19:1)	-0.902	-3.3275	<0.001	0.012	*

		LPE(20:3)	-0.705	-2.5908	0.010	0.060	.
		LPE(20:5)	-0.564	-2.092	0.037	0.064	.
		LPE(22:4)	-0.612	-2.3235	0.021	0.061	.
		LPE(22:5)	-0.581	-2.0987	0.037	0.064	.
		LPE(24:6)	-0.62	-2.3696	0.019	0.061	.
	Phosphatidyl-ethanolamines	PE(dO-38:9),PE(35:2),PE(O-36:2),PE(P-36:1)	-0.877	-3.1949	0.002	0.087	.
	Phosphatidyl-inositols	PI(34:2),PI(O-35:2),PI(P-35:1)	-0.765	-3.0105	0.003	0.017	*
		PI(34:3),PI(P-35:2)	-0.65	-2.3447	0.020	0.059	.
		PI(34:5)	-0.669	-2.455	0.015	0.059	.
		PI(36:3),PI(P-37:2)	-0.512	-1.9907	0.048	0.095	.
		PI(36:4)	-0.509	-2.0385	0.043	0.095	.
		PI(38:3)	-0.498	-1.8502	0.065	0.098	.
		PI(38:6)	-0.513	-1.8965	0.059	0.098	.
**PFOA**	Phosphatidyl-inositols	PI(40:5)	-0.879	-3.2103	0.002	0.018	*
**PFOS**	Phosphatidyl-gylcerolphosphates	PG(34:4)	-0.578	-1.9973	0.047	0.091	.
	Phosphatidyl-inositols	PI(34:5)	-0.815	-2.9819	0.003	0.038	*
		PI(40:5)	-0.726	-2.6555	0.008	0.051	.

Table S24. Table reporting pairwise FDR-adjusted contrasts conducted for semi-quantitative linear models with covariate adjustment for spatial blocks, NF stage, and genetic sex using MetaboAnalyst 6.0. Abbreviation for FDR-adjusted p-values are as follows: "*" ≤ 0.05; "." ≤ 0.1

**Treatment**	**Lipid Class**	**Tentative Lipid ID**	**Log(FC)**	**t-value**	***p***	***adj p***	**Sig**
**PFHxA**	Phosphatidyl-cholines	PC(30:2),PC(P-31:1)	0.72175	2.602	0.010	0.094	.
		PC(32:0),PC(O-33:0)	0.62704	2.3539	0.020	0.094	.
		PC(32:1),PC(O-33:1),PC(P-33:0)	0.49705	2.5473	0.012	0.094	.

	PC(32:2),PC(O-33:2),PC(P-33:1)	0.40942	2.5608	0.011	0.094	.
	PC(33:0),PC(O-34:0)	0.71182	2.6181	0.010	0.094	.
	PC(34:1),PC(O-35:1),PC(P-35:0)	0.69487	2.4567	0.015	0.094	.
	PC(34:2),PC(O-35:2),PC(P-35:1)	0.5711	2.5068	0.013	0.094	.
	PC(35:2),PC(O-36:2),PC(P-36:1)	0.60309	2.316	0.022	0.094	.
	PC(35:3),PC(O-36:3),PC(P-36:2)	0.56901	2.3035	0.023	0.094	.
	PC(36:3),PC(P-37:2)	0.45113	2.3108	0.022	0.094	.
	PC(36:4),PC(O-37:4)	0.54915	2.2768	0.024	0.097	.
	PC(36:5)	0.50138	2.4629	0.015	0.094	.
	PC(37:6),PC(O-38:6),PC(P-38:5)	0.71248	2.4941	0.014	0.094	.
	PC(38:3)	0.64646	2.3797	0.019	0.094	.
	PC(38:4)	0.65963	2.4011	0.018	0.094	.
	PC(38:5)	0.67271	2.8711	0.005	0.094	.
	PC(38:6)	0.64543	2.7251	0.007	0.094	.
	PC(39:7),PC(P-40:6),PC(38:0),PC(O-39:0)	0.77131	2.6422	0.009	0.094	.
	PC(40:5)	0.69714	2.3969	0.018	0.094	.
	PC(40:6)	0.78862	3.2087	0.002	0.094	.
	PC(40:7),PC(39:0),PC(O-40:0)	0.5689	2.4606	0.015	0.094	.
	PC(42:3)	0.66652	2.35	0.020	0.094	.
	PC(42:7),PC(41:0),PC(O-42:0)	0.78705	2.837	0.005	0.094	.
	PC(42:8),PC(41:1),PC(O-42:1),PC(P-42:0)	0.70492	2.3762	0.019	0.094	.
	PC(O-38:8),PC(36:1),PC(O-37:1),PC(P-37:0)	0.66707	2.2601	0.025	0.098	.
	PC(O-38:9),PC(36:2),PC(O-37:2),PC(P-37:1)	0.66058	2.7834	0.006	0.094	.
Sphingomyelins	SM(d16:1/20:1)	0.84357	3.3187	0.001	0.047	*

	Cholesterol ester	CE(15:1)	0.76826	2.6847	0.008	0.073	.
**PFHxS**	Phosphatidyl-cholines	PC(30:2),PC(P-31:1)	0.85104	3.0752	0.003	0.049	*
		PC(31:0),PC(O-32:0)	0.61352	2.4669	0.015	0.063	.
		PC(32:0),PC(O-33:0)	0.79536	2.9926	0.003	0.049	*
		PC(32:1),PC(O-33:1),PC(P-33:0)	0.64253	3.3005	0.001	0.049	*
		PC(32:2),PC(O-33:2),PC(P-33:1)	0.40638	2.5476	0.012	0.053	.
		PC(33:0),PC(O-34:0)	0.76002	2.8019	0.006	0.049	*
		PC(33:0),PC(O-34:0)	0.76002	2.8019	0.006	0.049	*
		PC(33:1),PC(O-34:1),PC(P-34:0)	0.81639	2.7396	0.007	0.050	*
		PC(33:2),PC(O-34:2),PC(P-34:1)	0.80062	2.8089	0.006	0.049	*
		PC(34:0),PC(O-35:0)	0.84242	2.9122	0.004	0.049	*
		PC(34:1),PC(O-35:1),PC(P-35:0)	0.81525	2.8889	0.004	0.049	*
		PC(34:2),PC(O-35:2),PC(P-35:1)	0.64247	2.8265	0.005	0.049	*
		PC(34:3),PC(P-35:2)	0.44887	2.3265	0.021	0.072	.
		PC(35:2),PC(O-36:2),PC(P-36:1)	0.68456	2.635	0.009	0.050	*
		PC(35:3),PC(O-36:3),PC(P-36:2)	0.76875	3.1193	0.002	0.049	*
		PC(35:4),PC(O-36:4),PC(P-36:3)	0.52748	2.1378	0.034	0.098	.
		PC(36:3),PC(P-37:2)	0.51785	2.6586	0.009	0.050	*
		PC(36:4),PC(O-37:4)	0.5916	2.4584	0.015	0.063	.
		PC(36:5)	0.5225	2.5725	0.011	0.053	.
		PC(36:8),PC(35:1),PC(O-36:1),PC(P-36:0)	0.69913	2.3532	0.020	0.070	.
		PC(37:4),PC(O-38:4),PC(P-38:3)	0.69259	2.3052	0.023	0.072	.
		PC(37:6),PC(O-38:6),PC(P-38:5)	0.81941	2.8751	0.005	0.049	*

	PC(37:7),PC(P-38:6),PC(36:0),PC(O-37:0)	0.69125	2.3159	0.022	0.072	.
	PC(38:3)	0.73275	2.7035	0.008	0.050	*
	PC(38:4)	0.65694	2.3968	0.018	0.069	.
	PC(38:5)	0.65036	2.7821	0.006	0.049	*
	PC(38:6)	0.63575	2.6903	0.008	0.050	*
	PC(38:7),PC(37:0),PC(O-38:0)	0.52903	2.7117	0.008	0.050	*
	PC(38:8),PC(37:1),PC(O-38:1),PC(P-38:0)	0.67879	2.403	0.018	0.069	.
	PC(38:9),PC(37:2),PC(O-38:2),PC(P-38:1)	0.66295	2.5726	0.011	0.053	.
	PC(39:6),PC(O-40:6),PC(P-40:5)	0.678	2.7593	0.007	0.094	.
	PC(39:7),PC(P-40:6),PC(38:0),PC(O-39:0)	0.68557	2.3539	0.020	0.070	.
	PC(40:5)	0.66465	2.2904	0.023	0.073	.
	PC(40:6)	0.7352	2.9982	0.003	0.049	*
	PC(40:7),PC(39:0),PC(O-40:0)	0.58819	2.5499	0.012	0.053	.
	PC(40:8),PC(39:1),PC(O-40:1),PC(P-40:0)	0.64543	2.2112	0.029	0.084	.
	PC(42:5)	0.85613	2.8786	0.005	0.049	*
	PC(42:8),PC(41:1),PC(O-42:1),PC(P-42:0)	0.7026	2.3739	0.019	0.070	.
	PC(O-38:8),PC(36:1),PC(O-37:1),PC(P-37:0)	0.65238	2.2154	0.028	0.084	.
	PC(O-38:9),PC(36:2),PC(O-37:2),PC(P-37:1)	0.69597	2.9392	0.004	0.049	*
Phosphatidyl-ethanolamines	PE(34:2),PE(O-35:2),PE(P-35:1)	0.41859	2.6128	0.010	0.029	*
	PE(35:3),PE(O-36:3),PE(P-36:2)	0.55193	2.1149	0.036	0.067	.
	PE(36:3),PE(P-37:2)	0.4826	2.7784	0.006	0.024	*
	PE(36:4),PE(O-37:4)	0.61402	2.9342	0.004	0.018	*

PE(36:5)	0.39276	2.5493	0.012	0.030	*
PE(36:6)	0.58944	2.6812	0.008	0.029	*
PE(37:4),PE(O-38:4),PE(P-38:3)	0.81953	3.4708	0.001	0.008	*
PE(37:5),PE(O-38:5),PE(P-38:4)	0.88263	3.3314	0.001	0.008	*
PE(37:6),PE(O-38:6),PE(P-38:5)	0.81382	3.3821	0.001	0.008	*
PE(38:3)	0.72325	2.5974	0.010	0.029	*
PE(38:4)	0.73071	2.5098	0.013	0.032	*
PE(38:5)	0.72046	3.4695	0.001	0.008	*
PE(38:6)	0.61333	3.5316	0.001	0.008	*
PE(38:7),PE(37:0),PE(O-38:0)	0.49247	2.5838	0.011	0.029	*
PE(38:9),PE(dO-40:9),PE(37:2),PE(O-38:2),PE(P-38:1)	1.1183	4.0546	0.000	0.004	*
PE(39:4),PE(O-40:4),PE(P-40:3)	0.71424	2.4616	0.015	0.032	*
PE(39:5),PE(O-40:5),PE(P-40:4)	0.82022	2.967	0.004	0.018	*
PE(39:6),PE(O-40:6),PE(P-40:5)	0.55266	2.5995	0.010	0.029	*
PE(40:10),PE(39:3), PE(O-40:3),PE(P-40:2)	0.70548	2.4782	0.014	0.032	*
PE(40:4)	0.72913	2.6323	0.009	0.029	*
PE(40:5)	0.80403	3.0231	0.003	0.017	*
PE(40:6),PE(dO-40:0)	0.69269	3.4345	0.001	0.008	*
PE(40:7),PE(39:0),PE(O-40:0)	0.58566	3.1124	0.002	0.014	*
PE(40:8),PE(39:1),PE(O-40:1),PE(P-40:0)	0.48579	1.9894	0.049	0.081	.
PE(40:9),PE(39:2),PE(O-40:2),PE(P-40:1)	0.5922	2.2535	0.026	0.052	.
PE(42:9),PE(41:2),PE(O-42:2),PE(P-42:1)	0.60433	2.1916	0.030	0.058	.
PE(O-38:8),PE(36:1),PE(O-37:1),PE(P-37:0)	0.5903	2.0768	0.040	0.071	.

		PE(O-38:9),PE(36:2),PE(O-37:2),PE(P-37:1)	0.63861	2.8509	0.005	0.021	*
		PE(O-40:9),PE(38:2),PE(P-39:1)	0.68123	2.4588	0.015	0.032	*
	Spingomyelin	SM(d16:0/20:0)	0.55655	2.551	0.012	0.070	.
		SM(d16:0/22:0)	0.66412	2.4232	0.017	0.070	.
		SM(d16:1/17:0)	0.67632	2.3525	0.020	0.070	.
		SM(d16:1/18:0)	0.46693	2.0673	0.040	0.089	.
		SM(d16:1/20:0)	0.67399	2.8692	0.005	0.070	.
		SM(d16:1/23:0)	0.66251	2.6012	0.010	0.070	.
		SM(d16:1/24:0)	0.83889	3.2447	0.001	0.060	.
		SM(d16:1/24:1)	0.54969	2.5744	0.011	0.070	.
		SM(d16:1/25:0)	0.70928	2.4582	0.015	0.070	.
		SM(d17:1/24:1)	0.59018	2.1421	0.034	0.086	.
		SM(d18:0/15:0)	0.64024	2.1444	0.034	0.086	.
		SM(d18:0/24:0)	0.63411	2.1687	0.032	0.086	.
		SM(d18:0/24:1)	0.67129	2.5704	0.011	0.070	.
		SM(d18:1/17:0)	0.62354	2.291	0.023	0.075	.
		SM(d18:1/19:0)	0.78572	2.7842	0.006	0.070	.
		SM(d18:1/24:1(15Z))	0.50767	2.1057	0.037	0.086	.
		SM(d18:2/20:1)	0.58584	2.1194	0.036	0.086	.
		SM(d18:2/21:0)	0.59885	2.3606	0.019	0.070	.
		SM(d18:2/24:1)	0.63173	2.4111	0.017	0.070	.
**PFOA**	Lysophosphatidy-choline	LPC(19:3)	0.89432	3.1346	0.002	0.051	.
	Lysophosphatidyl-ethanolamines	LPE(20:4)	0.69137	2.7498	0.007	0.044	*
		LPE(20:5)	0.8145	2.8897	0.004	0.044	*
		LPE(22:4)	0.72503	2.5085	0.013	0.049	*
		LPE(22:5)	0.58498	2.3774	0.019	0.049	*
		LPE(22:6)	0.51404	2.4446	0.016	0.049	*
	Phosphatidyl-choline	PC(39:6),PC(O-40:6),PC(P-40:5)	0.6492	2.6482	0.009	0.050	*

	PE(35:3),PE(O-36:3),PE(P-36:2)	0.85224	3.4117	0.001	0.021	*
	PE(36:3),PE(P-37:2)	0.43375	2.4849	0.014	0.062	.
	PE(36:4),PE(O-37:4)	0.48558	2.309	0.023	0.080	.
	PE(37:5),PE(O-38:5),PE(P-38:4)	0.71722	2.6937	0.008	0.058	.
	PE(37:6),PE(O-38:6),PE(P-38:5)	0.54832	2.2675	0.025	0.083	.
	PE(38:3)	0.61339	2.192	0.030	0.089	.
	PE(38:4)	0.78657	2.6884	0.008	0.058	.
	PE(38:5)	0.53868	2.5813	0.011	0.061	.
	PE(38:6)	0.39029	2.2363	0.027	0.084	.
	PE(38:9),PE(dO-40:9),PE(37:2),PE(O-38:2),PE(P-38:1)	0.8898	3.2104	0.002	0.021	*
	PE(39:5),PE(O-40:5),PE(P-40:4)	0.71653	2.5791	0.011	0.061	.
	PE(39:7),PE(P-40:6),PE(38:0),PE(O-39:0)	0.58364	2.4386	0.016	0.062	.
	PE(40:6),PE(dO-40:0)	0.57244	2.8243	0.005	0.055	.
	PE(40:7),PE(39:0),PE(O-40:0)	0.61916	3.2742	0.001	0.021	*
	PE(42:9),PE(41:2),PE(O-42:2),PE(P-42:1)	0.97644	3.5237	0.001	0.021	*
	PE(O-38:8),PE(36:1),PE(O-37:1),PE(P-37:0)	0.70777	2.4778	0.014	0.062	.
	PE(O-38:9),PE(36:2),PE(O-37:2),PE(P-37:1)	0.54967	2.4417	0.016	0.062	.
Phosphatidyl-serines	PS(38:4)	0.5733	2.6651	0.008	0.085	.
Sphingomyelins	SM(d16:1/17:0)	0.95614	3.4051	0.001	0.035	*
	SM(d16:1/20:0)	0.52604	2.2928	0.023	0.100	.
	SM(d16:1/23:0)	0.5447	2.1896	0.030	0.100	.
	SM(d16:1/24:0)	0.76327	3.0226	0.003	0.053	.
	SM(d16:1/25:0)	0.61389	2.1783	0.031	0.100	.
	SM(d17:1/24:1)	0.6886	2.5589	0.011	0.076	.
	SM(d17:1/26:1)	0.63885	2.2002	0.029	0.100	.

		SM(d18:0/15:0)	0.75286	2.5817	0.011	0.076	.
		SM(d18:0/24:0)	0.72054	2.5231	0.013	0.076	.
		SM(d18:1/17:0)	0.59253	2.229	0.027	0.100	.
		SM(d18:1/19:0)	0.80957	2.9371	0.004	0.053	.
		SM(d18:2/21:0)	0.69865	2.8196	0.005	0.057	.
		SM(d18:2/24:1)	0.60887	2.3793	0.019	0.097	.
**PFOS**	Lysophosphatidyl-choline	LPC(16:1),LPC(P-17:0)	0.6492	2.4668	0.014	0.087	.
		LPC(17:1),LPC(O-18:1),LPC(P-18:0)	0.73178	2.5147	0.013	0.087	.
		LPC(19:3)	0.6808	2.3616	0.019	0.087	.
		LPC(20:2),PC(O-20:2)	0.64918	2.3951	0.017	0.087	.
		LPC(20:3)	0.55742	2.1877	0.030	0.097	.
		LPC(22:4)	0.64738	2.3096	0.022	0.087	.
		LPC(22:5)	0.58892	2.2824	0.023	0.087	.
		LPC(24:1)	0.64933	2.3513	0.020	0.087	.
		LPE(20:1)	0.66134	2.1953	0.030	0.064	.
		LPE(20:3)	0.6475	2.7643	0.006	0.042	*
		LPE(20:4)	0.61351	2.3868	0.018	0.059	.
		LPE(20:5)	0.65543	2.2746	0.024	0.063	.
		LPE(22:5)	0.69861	2.7772	0.006	0.042	*
		LPE(22:6)	0.55424	2.5781	0.011	0.047	*
	Phosphatidyl-ethanolamines	PE(20:0),LPE(21:0)	0.59659	2.0169	0.046	0.095	.
		PE(31:0),PE(O-32:0)	0.73253	2.4885	0.014	0.054	.
		PE(34:2),PE(O-35:2),PE(P-35:1)	0.3245	1.9913	0.049	0.097	.
		PE(36:3),PE(P-37:2)	0.43427	2.4579	0.015	0.054	.
		PE(36:5)	0.38463	2.4543	0.015	0.054	.
		PE(36:6)	0.54958	2.4576	0.015	0.054	.
		PE(37:4),PE(O-38:4),PE(P-38:3)	0.65105	2.7106	0.008	0.048	*
		PE(37:5),PE(O-38:5),PE(P-38:4)	0.79414	2.9467	0.004	0.038	*
		PE(38:3)	0.6903	2.4371	0.016	0.054	.
		PE(38:4)	0.66574	2.248	0.026	0.066	.

	PE(38:5)	0.5876	2.7818	0.006	0.045	*
	PE(38:6)	0.49044	2.7762	0.006	0.045	*
	PE(38:7),PE(37:0),PE(O-38:0)	0.40338	2.0806	0.039	0.090	.
	PE(38:9),PE(dO-40:9),PE(37:2),PE(O-38:2),PE(P-38:1)	0.62267	2.2195	0.028	0.067	.
	PE(39:5),PE(O-40:5),PE(P-40:4)	0.63075	2.243	0.027	0.066	.
	PE(39:6),PE(O-40:6),PE(P-40:5)	0.6696	3.0962	0.002	0.038	*
	PE(39:7),PE(P-40:6),PE(38:0),PE(O-39:0)	0.90747	3.746	0.000	0.013	*
	PE(40:10),PE(39:3), PE(O-40:3),PE(P-40:2)	0.58589	2.0233	0.045	0.095	.
	PE(40:6),PE(dO-40:0)	0.60524	2.9501	0.004	0.038	*
	PE(40:7),PE(39:0),PE(O-40:0)	0.58708	3.0671	0.003	0.038	*
	PE(40:9),PE(39:2),PE(O-40:2),PE(P-40:1)	0.67074	2.5092	0.013	0.054	.
	PE(42:9),PE(41:2),PE(O-42:2),PE(P-42:1)	0.63609	2.2678	0.025	0.066	.
	PE(O-38:8),PE(36:1),PE(O-37:1),PE(P-37:0)	0.6533	2.2596	0.025	0.066	.
	PE(O-38:9),PE(36:2),PE(O-37:2),PE(P-37:1)	0.51182	2.2462	0.026	0.066	.
	PE(O-40:9),PE(38:2),PE(P-39:1)	0.69104	2.452	0.016	0.054	.
Sphingomyelins	SM(d16:0/22:0)	0.68082	2.517	0.013	0.088	.
	SM(d16:1/23:0)	0.72026	2.8654	0.005	0.046	*
	SM(d16:1/24:0)	0.83622	3.2772	0.001	0.046	*
	SM(d16:1/24:1)	0.49961	2.3708	0.019	0.088	.
	SM(d16:1/25:0)	0.86272	3.0295	0.003	0.046	*
	SM(d17:1/24:1)	0.76629	2.8181	0.005	0.046	*
	SM(d17:1/26:1)	0.65997	2.2494	0.026	0.099	.

		SM(d18:0/24:0)	0.85766	2.9721	0.003	0.046	*
		SM(d18:1/17:0)	0.64549	2.403	0.017	0.088	.
		SM(d18:1/19:0)	0.63711	2.2875	0.023	0.099	.
		SM(d18:1/25:0)	0.67969	2.459	0.015	0.088	.
**MIX**	Diglycerides	DG(32:0)_C16:0	0.38377	2.3011	0.023	0.072	.
		DG(32:1)_C16:0	0.45729	3.1832	0.002	0.036	*
		DG(32:1)_C16:1	0.40465	2.8568	0.005	0.036	*
		DG(32:2)_C16:1	0.39405	2.5915	0.011	0.046	*
		DG(33:1),DG(O-34:1)_C16:1	0.52901	2.6625	0.009	0.046	*
		DG(33:2)_C16:1	0.53372	2.5792	0.011	0.046	*
		DG(34:1)_C16:0	0.43327	3.0094	0.003	0.036	*
		DG(34:1)_C18:1	0.4041	2.9139	0.004	0.036	*
		DG(34:2)_C16:0	0.43777	2.8571	0.005	0.036	*
		DG(34:2)_C16:1	0.47859	2.3651	0.019	0.067	.
		DG(34:2)_C18:2	0.46123	2.8328	0.005	0.036	*
		DG(34:3)_C16:1	0.41063	2.5726	0.011	0.046	*
		DG(36:3)_C18:1	0.38706	2.1615	0.032	0.095	.
		DG(O-38:9),DG(36:2)_C18:1	0.38304	2.4027	0.018	0.066	.
	Triglycerides	[TG(46:1)]_FA14:0	0.30559	1.9113	0.058	0.086	.
		[TG(46:1)]_FA16:1	0.33101	2.0292	0.044	0.086	.
		[TG(48:2)]_FA14:0	0.31938	2.0941	0.038	0.086	.
		[TG(48:2)]_FA16:0	0.27022	1.6778	0.095	0.100	.
		[TG(48:2)]_FA16:1	0.27848	1.7256	0.086	0.099	.
		[TG(48:8),TG(47:1)]_FA16:1	0.32181	2.007	0.047	0.086	.
		[TG(49:8),TG(48:1)]_FA16:0	0.29708	1.8274	0.070	0.086	.
		[TG(50:3)]_FA16:0	0.29805	1.8189	0.071	0.086	.
		[TG(50:3)]_FA16:1	0.29624	1.8852	0.061	0.086	.
		[TG(50:8),TG(49:1)]_FA16:0	0.29862	1.9808	0.049	0.086	.
		[TG(50:9),TG(49:2)]_FA16:1	0.29172	1.9096	0.058	0.086	.
		[TG(51:8),TG(50:1)]_FA16:0	0.32113	2.0163	0.046	0.086	.

[TG(51:8),TG(50:1)]_FA18:1	0.3211	2.0319	0.044	0.086	.
[TG(51:9),TG(50:2)]_FA16:0	0.28648	1.8143	0.072	0.086	.
[TG(51:9),TG(50:2)]_FA16:1	0.30509	1.945	0.054	0.086	.
[TG(51:9),TG(50:2)]_FA18:1	0.30276	1.9501	0.053	0.086	.
[TG(51:9),TG(50:2)]_FA18:2	0.27769	1.6786	0.095	0.100	.
[TG(52:4)]_FA18:2	0.31533	1.962	0.052	0.086	.
[TG(52:5)]_FA20:4	0.42389	2.5275	0.013	0.086	.
[TG(53:10),TG(52:3)]_FA18:1	0.31385	2.0459	0.042	0.086	.
[TG(53:9),TG(52:2)]_FA18:1	0.32699	2.1164	0.036	0.086	.
[TG(54:5)]_FA18:1	0.34007	2.1177	0.036	0.086	.
[TG(56:7),TG(55:0)]_FA22:6	0.50135	2.307	0.022	0.086	.
